# Mechanism of biofilm-mediated stress resistance and lifespan extension in *C. elegans*

**DOI:** 10.1038/s41598-017-07222-8

**Published:** 2017-08-02

**Authors:** Olga Smolentseva, Ivan Gusarov, Laurent Gautier, Ilya Shamovsky, Alicia S. DeFrancesco, Richard Losick, Evgeny Nudler

**Affiliations:** 1 Department of Biochemistry and Molecular Pharmacology, New York University School of Medicine, New York, NY 10016 USA; 2 Department of Molecular and Cellular Biology, Harvard University, Cambridge, Massachusetts 02138 USA; 3 Howard Hughes Medical Institute, New York University School of Medicine, New York, NY 10016 USA

## Abstract

Bacteria naturally form communities of cells known as biofilms. However the physiological roles of biofilms produced by non-pathogenic microbiota remain largely unknown. To assess the impact of a biofilm on host physiology we explored the effect of several non-pathogenic biofilm-forming bacteria on *Caenorhabditis elegans*. We show that biofilm formation by *Bacillus subtilis, Lactobacillus rhamnosus* and *Pseudomonas fluorescens* induces *C. elegans* stress resistance. Biofilm also protects against pathogenic infection and prolongs lifespan. Total mRNA analysis identified a set of host genes that are upregulated in response to biofilm formation by *B. subtilis*. We further demonstrate that *mtl-1* is responsible for the biofilm-mediated increase in oxidative stress resistance and lifespan extension. Induction of *mtl-1* and *hsp-70* promotes biofilm-mediated thermotolerance. *ilys-2* activity accounts for biofilm-mediated resistance to *Pseudomonas aeruginosa* killing. These results reveal the importance of non-pathogenic biofilms for host physiology and provide a framework to study commensal biofilms in higher organisms.

## Introduction

Bacteria colonize their hosts, as they do other natural surfaces, predominantly as biofilms^1, 2^. In a biofilm, the encased community of bacterial cells is held in a self-produced extracellular matrix^1^ that serves as a physical and chemical diffusion barrier, but allows for cell differentiation and specialization^3, 4^. Biofilms thus offer bacteria strong competitive advantages under various environmental challenges^2^. For example, biofilms render bacteria less susceptible to host immunity and to antibiotics^5^. As biofilms contribute to the recalcitrance of chronic infections, they have been studied primarily with respect to their effects on human pathogens, such as *Pseudomonas aeruginosa* ^5^, whereas the impact of non-pathogenic biofilms on the host remains largely unknown.

The enormous complexity of the mammalian microbiota presents significant challenges to deciphering the specific mechanisms by which it influences host health and fitness. It has been difficult to establish whether changes that occur in microbiota are the cause or effect of concomitant pathologies and age-related transformations in well-controlled mouse models, and it is even more difficult in human subjects^6, 7^. Moreover, sessile bacteria are notoriously more difficult to study than their planktonic counterparts.

*C. elegans* represents a robust model for the study of host-microbiota interaction, as it is easily manipulated in the laboratory, has a short lifespan, is genetically tractable, and its colonization by bacteria can be controlled^8^. We used a defined model system consisting of *C. elegans* and *B. subtilis* to study bacterial biofilm-host interaction. In its natural habitat, such as decomposing plant material, *C. elegans* proliferates on live, actively metabolizing bacteria, including *Bacillus* ^9–11^ (see Supplemental Information), which serve not only as a food source but also contribute to the worm’s health and lifespan^12–15^. In the same natural environment *Bacillus* forms biofilms that have been well characterized genetically and biochemically^16, 17^. Moreover, it was recently reported that *C. elegans* fed on spores of undomesticated *B. subtilis* live longer and exhibit higher stress resistance as compared to worms fed on spores of a domesticated strain^18^. Here we demonstrate that biofilm formation by *B. subtilis* improves *C. elegans* resistance to infection and stress and prolongs lifespan. Using biofilm-deficient mutants of *B. subtilis*, we also identify specific *C. elegans* genes that confer biofilm-mediated phenotypes. Additionally, we were able to visualize biofilm formed by *B. subtilis* in *C. elegans* intestine and show that other non-pathogenic bacterial biofilms, such as those produced by *L. rhamnosus* and *P. fluorescens*, also promote stress resistance in worms.

## Results

### Biofilm formation by B. subtilis augments C. elegans resistance to stress

**Figure 1:**
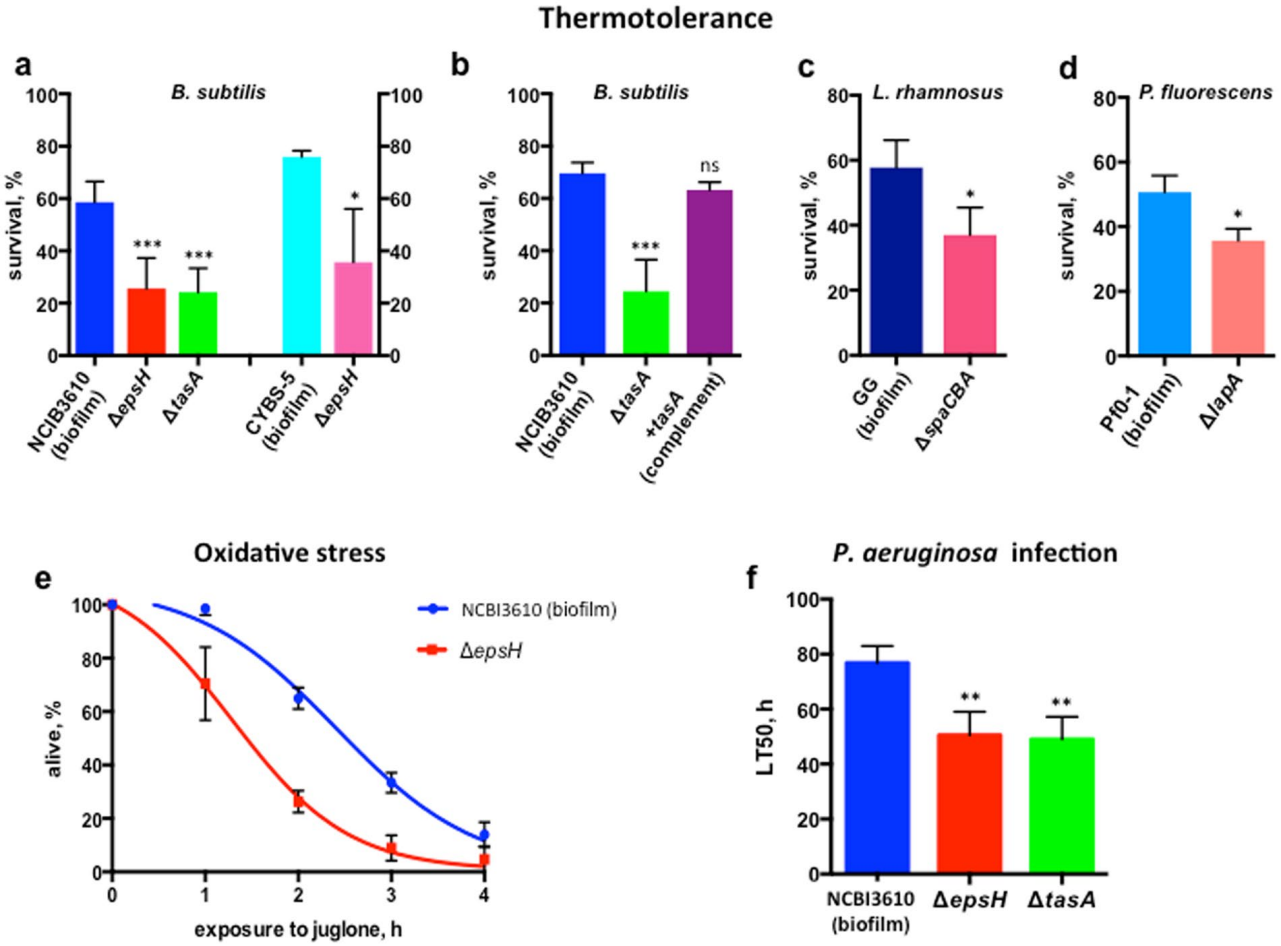
Biofilm enhances *C. elegans* stress resistance. Each graph represents mean values ± SD from at least free independent biological replicates. Each biological replicate was performed with at least 60 worms per condition. (**a**) *B. subtilis* biofilm increases *C. elegans* thermotolerance. left panel, Day-five-adult worms grown on *B. subtilis* NCBI3610 (biofilm) or its biofilm-deficient mutants, Δ*epsH* and Δ*tasA*, were subjected to heat shock at 33 °C for 3 hours. Surviving animals were scored after 20 hours of recovery at 20 °C. Mean values ± SD are plotted, n = 5. right panel, *C. elegans* were grown on *B. subtilis* CYBS-5 (biofilm) or its biofilm-deficient derivative (Δ*epsH*) until day 5 of adulthood and subjected to heat shock at 33 °C for 3 hours. Surviving animals were scored after 20 hours of recovery at 20 °C. T-test p-value = 0.0270, n = 3. (**b**) Complementation of *B. subtilis tasA* deficiency restores biofilm-mediated enhancement of *C. elegans* thermotolerance. Worms were grown on either biofilm-forming NCBI3610 *B. subtilis* (biofilm), its biofilm-deficient derivative (Δ*tasA*), or ∆*tasA* complimented with a copy of *tasA* inserted at a distal locus (+tasA (complement)). Five-day old adult worms were heat shocked and scored as described in (**a**), n = 3. (**c**) The *Lactobacillus rhamnosus* biofilm enhances worms resistance to elevated temperatures. *C. elegans* were grown on *Lactobacillus rhamnosus* GG (biofilm) or its biofilm-deficient derivative (Δ*spaCBA*) until day 5 of adulthood and subjected to heat shock at 36 °C for 3 hours. After 20 hours of recovery at 20 °C it was unclear if animals were dead due to residual movement, thus surviving animals were scored after 40 hours of recovery at 20 °C. T-test p-value = 0.0389, n = 3. (**d**) The *Pseudomonas fluorescens* biofilm promotes worm heat shock-resistance. *C. elegans* were grown on *Pseudomonas fluorescens* Pf0-1(biofilm) or its biofilm-deficient derivative (Δ*lapA*) until day 5 of adulthood and subjected to heat shock at 35 °C for 4 hours. Surviving animals were scored after 20 hours of recovery at 20 °C. T-test p-value = 0.0144, n = 3. (**e**) The biofilm renders *C. elegans* more resistant to oxidative stress. Five-day old *C. elegans*, grown on NCBI3610 (biofilm) or its biofilm-deficient derivative (Δ*epsH*) were transferred to plates supplemented with juglone (90 μM) and scored for survival every hour. For statistical data see Supplementary Table S1, n = 4. (**f**) *B. subtilis* biofilm protects against pathogenic bacteria. Five-day old worms fed NCBI3610 (biofilm) or its biofilm-deficient derivatives, Δ*epsH* and Δ*tasA*, were transferred to *P. aeruginosa* PA14 seeded plates and scored for survival every 6 hours. LT50, the time at which 50% of animals were scored as dead, was taken from Kaplan-Meier survival curves (median survival data). For the log-rank test p-values see Supplementary Table S2, n = 6.

The ability to withstand stress usually indicates that an organism is in a healthy state. Thus, to investigate the effect of *B. subtilis* biofilm formation on *C. elegans* physiology we first examined thermotolerance of worms fed the undomesticated *B. subtilis* isolate NCBI3610 and its biofilm-deficient derivatives, Δ*epsH* and Δ*tasA*, each of which lacks a different extracellular matrix component (exopolysaccharide and amyloid-like protein fibers, respectively) essential for biofilm formation^19, 20^. The use of two different biofilm-deficient strains eliminates the indirect effect of each individual mutant on heat-shock resistance. Bacterial colonization of *C. elegans* reaches its maximum by day 5 of adulthood^21^, therefore we subjected day-5-adults grown on the described *B. subtilis* strains to lethal heat shock at 33 °C. Biofilm-forming *B. subtilis* significantly improved the animals’ thermotolerance as compared to biofilm-deficient bacteria (Fig. 1a). Interestingly, when worms were subjected to lethal heat-shock at earlier stages of adulthood, we did not observe any significant biofilm-dependent survival advantages (Supplementary Fig. S1a,b), indicating that the biofilm’s beneficial effect requires time for the biofilm to actually form in the animal’s intestine. Complementation of the Δ*tasA* mutation with a copy of *tasA* inserted at a distal locus (*amyE*) restored biofilm formation and also increased the *C. elegans* stress resistance to the level observed with wild-type bacteria (Fig. 1b).

To substantiate our results we analyzed another natural biofilm-forming isolate of *B. subtilis*, CYBS-5. Similar to the experiment with NCBI3610, worms were more susceptible to heat stress when grown on biofilm-deficient derivative of CYBS-5 (Fig. 1a, right panel), suggesting that beneficial effects of the *B. subtilis* biofilm were not specific to any particular bacterial genetic background.

To address if the beneficial effect of biofilm is a unique property of *B, subtilis* or is also a feature of other non-pathogenic bacteria, we studied thermotolerance of *C. elegans* fed on another Gram-positive bacterium *Lactobacillus rhamnosus* GG and its biofilm-deficient counterpart (Δ*spaCBA*) (Fig. 1c) and the Gram-negative bacterium *Pseudomonas fluorescens* Pf0-1 and its biofilm-deficient mutant (Δ*lapA*) (Fig. 1d). Both *Lactobacillus and Pseudomonas* were found in the *C. elegans* natural microbiome^10, 11, 22^. *Lactobacillus rhamnosus* were shown to be beneficial for worms^23, 24^ and its biofilm was recently described^25^. *Pseudomonas fluorescens* Pf0-1 in standard growth conditions is also non-pathogenic for *C. elegans* ^26^ and its biofilm formation has been studied in detail^27, 28^. We found that *L. rhamnosus* and *P. fluorescens* biofilms also significantly promote *C. elegans* heat-resistance (Fig. 1c and d).

To further investigate the potential of a biofilm to improve *C. elegans* stress resistance, we assayed the animals’ survival after exposure to the oxidative agent juglone. We found that day-5-adults grown on biofilm-forming *B. subtilis* exhibited much greater resistance to oxidative stress compared to animals fed on biofilm-deficient bacteria (Fig. 1e and Supplementary Table S1). These results demonstrate that the biofilm produced by non-pathogenic bacterial is beneficial for host stress resistance.

### Biofilm formation by B. subtilis renders C. elegans resistant to pathogenic infection

Commensal bacteria are known to protect mammalian hosts from colonization by opportunistic pathogens^29^. To determine whether biofilm-producing *B. subtilis* can also reduce susceptibility to pathogenic infection, we examined the survival of *C. elegans* exposed to *P. aeruginosa*. We used the slow killing assay in which worms die from intestinal pathogenic colonization^26^. We grew worms on *B. subtilis* until day 5 of adulthood and then transferred them to agar plates seeded with *P. aeruginosa* PA14. Worms that were fed biofilm-forming bacilli displayed a much greater resistance to *P. aeruginosa* infection than did those that were fed biofilm-deficient strains (Fig. 1f and Supplementary Table S2).

Recently, it was suggested that spores of biofilm-forming *B. subtilis* can germinate and colonize the *C. elegans* intestine^18^. Also another wild isolate of *B. subtilis* GS67 can accumulate in *C. elegans* gut and protect animals from Gram-positive infection^12^. To determine whether the protective effect of biofilm-forming bacteria against lethal infection by pathogenic *P. aeruginosa* was due to their superior intestinal retention, we compared gut colonization by wild-type and biofilm-deficient *B. subtilis*, and found, by either colonization or guillotine assays, no significant difference in the number of cells of each *B. subtilis* strain in the gut lumen (Supplementary Fig. S2a and b). These results suggest that commensal biofilm-producing *B. subtilis* may not compete directly with pathogenic *Pseudomonas* in the *C. elegans* gut, but rather mobilize host defense system(s) against the pathogen.

### Biofilm formation by B. subtilis extends the C. elegans lifespan

**Figure 2:**
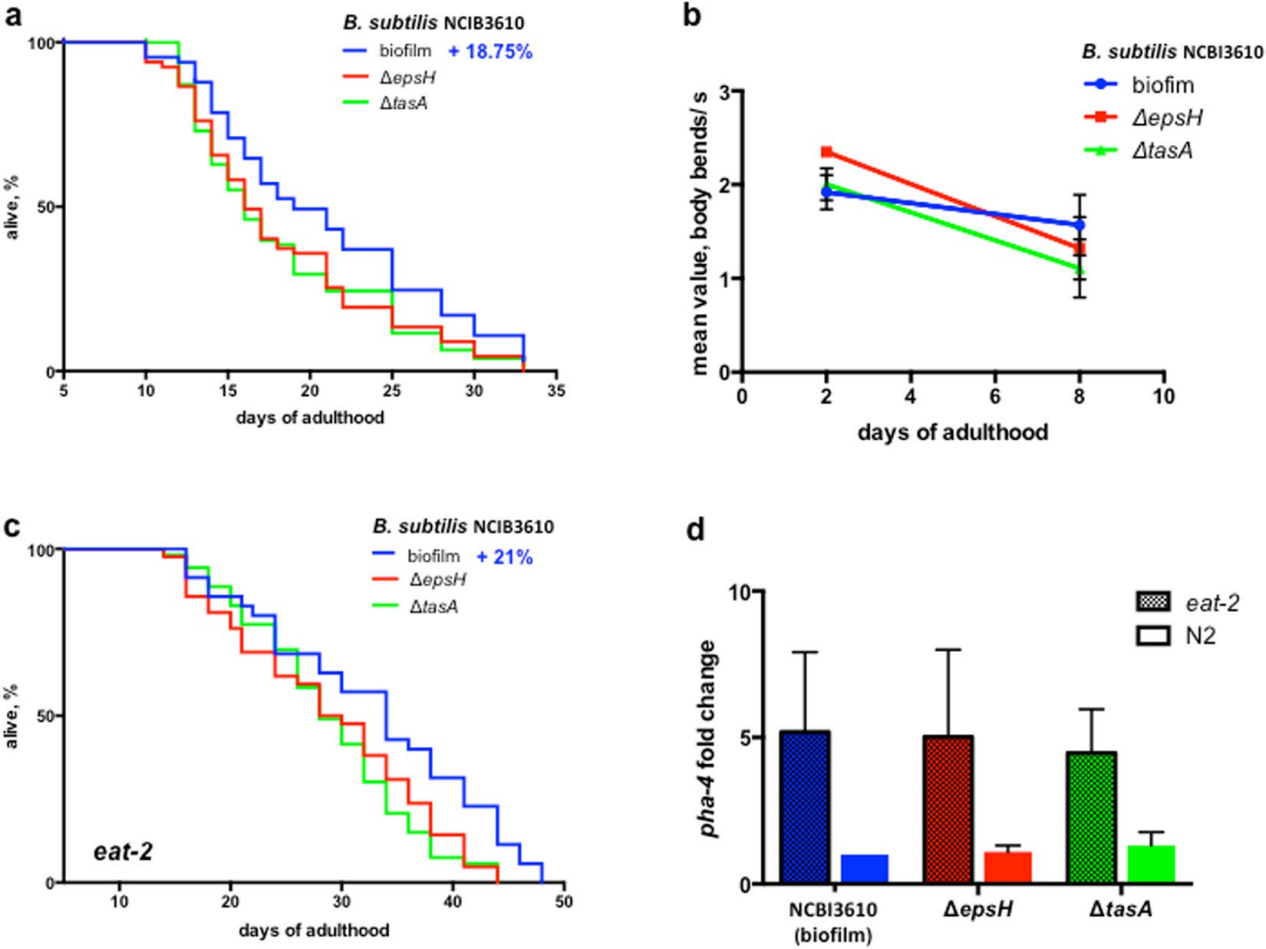
*B. subtilis* biofilm extends *C. elegans* lifespan independently of caloric restriction. (**a**) *C. elegans* N2 were fed either biofilm-forming *B. subtilis* NCBI3610 (biofilm) or its biofilm-deficient derivatives (Δ*epsH* or Δ*tasA*). The graph is representative of three independent biological replicates. Median lifespan, days: biofilm–19; Δ*epsH*–16; Δ*tasA*–16. The log-rank test p-value for each experiment is ≤0.05. Also see Supplementary Table S3. (**b**) Worms fed on biofilm-producing *B. subtilis* exhibit slower age-associated decline in motility. The graph represents linear regression of the median motility values of day-2 and day-8-adults grown of biofilm-forming (biofilm) or biofilm-deficient (Δ*epsH*, Δ*tasA*) *B. subtilis*. The median values were calculated as described in Supplemental Fig. S3d and mean values ± SD from 3 independent experiments are plotted. (**c**) The biofilm prolongs lifespan of dietary restricted worms DA1116 (*eat-2*). Median lifespan, days: biofilm–34; Δ*epsH*–29; Δ*tasA*–28. The graph is representative of three independent biological replicates. The log-rank test p-value is ≤0.05, Supplementary Table S3. (**d**) *pha-4* is induced in response to dietary restriction, but not by the *B. subtilis* biofilm. RT-PCR analysis of *pha-4* expression in *C. elegans eat-2* (dashed bars) and N2 (empty bars) fed biofilm-forming (biofilm) and biofilm deficient (Δ*epsH*, Δ*tasA*) *B. subtilis* strains. Mean ± SD from three independent experiments are plotted, n = 100 per experiment per condition. One-way ANOVA: *p*-value = 0.6696 for N2 worms; *p*-value = 0.1945 for *eat-2* worms.

Higher resistance to stress and pathogens often correlate with lifespan extension^14, 30^. To examine the potential benefit of the *B. subtilis* biofilm we measured its effect on *C. elegans* lifespan. The biofilm-deficient Δ*epsH* and Δ*tasA* mutants reduced median lifespan of *C. elegans* on average by, respectively, 20.5% and 15.1% as compared with the biofilm-producing parental strain (Fig. 2a and Supplementary Fig. S3a and b and Supplementary Table S3; Supplementary Discussion). Other physiological aspects of *C. elegans*, such as post-embryonic development and rate of egg production were not significantly affected (Supplementary Fig. S3c). Complementation of the Δ*tasA* mutation with a wild type copy of *tasA* restored *C. elegans* longevity to a wild-type biofilm level (Supplementary Fig. S3b and Supplementary Table S3), confirming that the bacterial effect on the worms’ lifespan was biofilm-specific.

To assess the impact of the biofilm on *C. elegans* healthspan we also studied its effect on motility. The rate of decline in motor activity and longevity are inversely correlated in worms^31–33^. We therefore measured the rate of decline in motility of animals grown on biofilm-forming and biofilm-deficient *B. subtilis* between day 2 and 8 of adulthood (Fig. 2b and Supplementary Fig. S3d). To avoid a possible bacterial strain-specific behavioral bias affecting *C. elegans* locomotion on the plate (either with or without food) we used a thrashing assay to study worm motility (Supplementary Fig. S3d). We found that biofilm formation slows down the age-associated decline in the *C. elegans* thrashing rate (Fig. 2b). These results, taken together with the beneficial effects of the biofilm on worm stress-resistance and lifespan, suggest that biofilm formation by a non-pathogenic bacteria improves the *C. elegans* healthspan.

### Biofilm-mediated effects do not entail dietary restriction

The nutritional status of bacteria as a food source is an important parameter that affects worm physiology^15, 34^. If biofilm formation diminishes the dietary value of *B. subtilis* cells, feeding *C. elegans* wild-type bacilli may induce a calorie restriction-like response, which is known to enhance lifespan and stress resistance^35, 36^. Moreover, domesticated *B. subtilis* 168 sporulates very efficiently on NGM, making it a poor nutritional source for *C. elegans* ^13, 37^. To address the nutritional issue, we first determined that the sporulation rates of undomesticated wild-type and its biofilm-deficient derivative are similar: both maintained over 70% vegetative bacteria under conditions used in our experiments (Supplementary Fig. S4a). We next studied the lifespan of an *eat-2* genetic mimetic of dietary restricted worms (DA1116). Biofilm-deficient *B. subtilis* decreased the lifespan of *eat-2* worms to about the same extent as that of N2 worms (Fig. 2c, Supplementary Table S3), arguing that caloric restriction is not a potential cause of biofilm-mediated phenotypes. We also showed that the mRNA levels of the *pha-4* transcription factor, which increases upon dietary restriction^38^, do not change in response to the biofilm (Fig. 2d). Moreover, the biofilm did not affect post-embryonic development (Supplementary Fig. S3c), animal size (Supplementary Fig. S4b and Supplementary Table S4) or the rate of pharyngeal pumping (Supplementary Fig. S4c). Together these results argue that the worms do not undergo caloric restriction in response to biofilm-forming *B. subtilis*.

### The biofilm exerts its effects from within the C. elegans intestinal lumen

**Figure 3:**
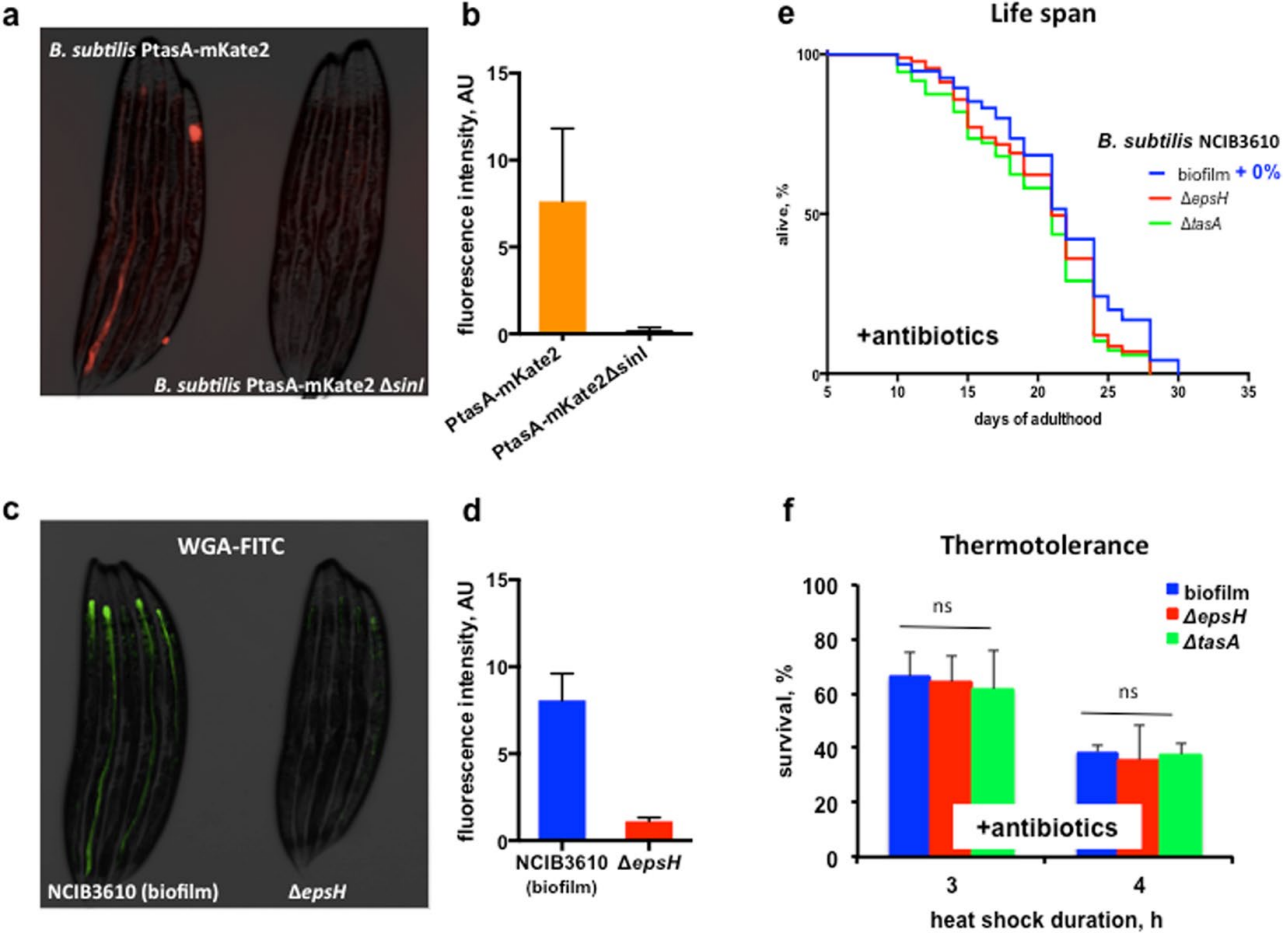
Beneficial effects likely require intestinal biofilm production. (**a**) Induction of biofilm matrix gene expression in the *C. elegans* intestine. Representative image of worms grown on the indicated *B. subtilis* strains were visualized at day 5 of adulthood. The red fluorescent signal indicates *tasA* expression in *B. subtilis* cells. (**b**) Fluorescence quantification of five-day old adults (n = 15) grown on corresponding bacterial strain. (**c**) Biofilm matrix exopolysaccharides are present in the *C. elegans* intestinal lumen. Representative image of FITC-conjugated wheat germ agglutinin (WGA-FITC) stained *C. elegans* grown on the indicated *B. subtilis* strains at day 5 of adulthood. Green fluorescence indicates biofilm matrix. (**d**) Fluorescence quantification of adult worms (n = 15) grown on corresponding bacterial strains until day 5 of adulthood and stained with WGA-FITC. Error bars show means ± SD from three independent experiments. (**e**) The anti-aging effect of biofilm requires live metabolizing bacteria. *B. subtilis* strains were grown on NGM plates overnight at 20 °C and then treated with a mixture of antibiotics (100 μg/ml kanamycin and 500 μg/ml carbenicillin). The graph is representative of three independent biological replicates. Median lifespan, days: biofilm–21; Δ*epsH*–21; Δ*tasA*–21. The log-rank test *p*-value for each experiment is >0.05. Supplementary Table S3. (**f**) Biofilm-induced thermotolerance requires live bacteria. *B. subtilis* lawns were treated with a mixture of antibiotics (100 μg/ml kanamycin and 500 μg/ml carbenicillin) prior to transferring *C. elegans* to them. Five-day old adult worms were heat shocked as in (**a**) for the indicated period of time. Each experiment includes at least 60 worms per condition per experiment. Mean values ± SD are plotted, n = 5. One-way ANOVA: p-value = 0.8759 (3 h), *p*-value = 0.9310 (4 h).

To confirm that *B. subtilis* was forming biofilm inside *C. elegans* we first used a strain that expresses the red fluorescent protein mKate2 under the control of a biofilm-specific promoter (*P*_*tasA*_-mKate2). Worms grown on *B. subtilis P*_*tasA*_-mKate2 exhibited red fluorescence in their intestinal lumen (Fig. 3a), indicating that biofilm matrix genes were being expressed. Moreover, mutation in the upstream regulator of biofilm synthesis *sinI* (*B. subtilis PtasA*-mKate2 Δ*sinI)* completely abrogated fluorescence in the intestine, demonstrating the specificity of the fluorescent signal to the induction of extracellular matrix genes inside *C. elegans* (Fig. 3a and b). To visualize biofilm formation, we also stained for biofilm exopolysaccharide in the gut of 5-day old worms with FITC-conjugated wheat germ agglutinin (WGA-FITC), a lectin. WGA-FITC was previously shown to bind to exopolysaccharides produced by *Yersinia pestis, Yersinia pseudotuberculosis*, and *Staphylococcus epidermidis* biofilms formed in association with *C. elegans* ^39, 40^. Also, WGA specifically binds to poly-N-acetylglucosamine, which is the major constituent of the *B. subtilis* biofilm matrix^41^. We therefore used it to label *B. subtilis* biofilm within *C. elegans*. As shown in Fig. 3c and d, WGA-FITC produced a strong signal in the presence of *B. subtilis* biofilms. Stained exopolysaccharides were detected only in the intestine of the worms fed with wild type *B. subtilis*, while in worms grown on Δ*epsH* we observed only minor intestinal staining due to the binding of WGA-FITC to worms’ glycocalyx^42^, and possibly due to weak binding to the *B. subtilis* cell wall.

To obtain independent visual support for biofilm formation in *C. elegans*, we examined worms fed with biofilm-producing *B. subtilis* (biofilm) or its Δ*epsH* mutant by electron microscopy and found vegetative cells surrounded by a dense matrix only in animals fed on biofilm-producing *B. subtilis* (Supplementary Fig. 5). Taken together, our data demonstrate that *B. subtilis* forms biofilms in the *C. elegans* intestine.

To determine whether the observed beneficial effects of the biofilm require live, metabolizing bacteria, we treated plated bacteria with a mix of antibiotics to eliminate vegetative cells, leaving previously-synthesized components of the biofilm intact (see Supplementary Discussion). Elimination of live bacteria abrogated all the positive effects of the biofilm on lifespan and thermotolerance (Fig. 3e and f and Supplementary Table S3). These results argue that it is not the extracellular matrix *per se*, but the metabolic state of biofilm-forming bacteria that benefits the worms.

### MTL-1 induction accounts for biofilm-mediated longevity and stress resistance

To understand the mechanism underlying the effects of the biofilm on worm physiology we determined the changes it causes in *C. elegans* gene transcription. Total mRNA sequencing revealed that a small group of *C. elegans* genes were differentially expressed in response to biofilm-forming vs. biofilm-deficient *B. subtilis* (Supplementary Table S5), demonstrating a specific response to the biofilm. To focus on *bona fide* biofilm-dependent changes, we assessed mRNA levels of identified genes in worms grown on the two biofilm-deficient mutants versus those in worms grown on wild-type *B. subtilis* (Supplementary Fig. S6). We also focused only on those genes that showed the same reproducible changes in response to both biofilm-deficient mutants used in our study (Supplementary Fig. S6, marked with asterisks). Based on supportive evidence from Real-Time PCR analyses and the relevance to observed *C. elegans* phenotypes, we selected four genes, *mtl-1*, *ilys-2*, *F44E5.4* and *F44E5.5* for in-depth analysis (Table 1) (see Supplementary Discussion).

**Table 1:** Induction of biofilm-responsive genes in *C. elegans*.

Gene	Proposed function	mRNA fold change		
		RNA-seq biofilm vs Δ*epsH*	RT-PCR biofilm vs Δ*epsH*	RT-PCR biofilm vs Δ*tasA*
*mtl-1*	metallothionein	1.47	1.60 ± 0.37	1.80 ± 0.54
*ilys-2*	invertebrate lysozyme	1.72	1.35 ± 0.05	1.72 ± 0.1
F44E5.5 (*hsp70*)	Inducible heat-shock protein 70	2.39	1.31 ± 0.01	1.44 ± 0.16
F44E5.4 (*hsp70*)		2.41	1.35 ± 0.07	1.31 ± 0.04

The table shows a list of genes whose expression changed (q-value < 0.05) in *C. elegans* fed biofilm-forming *B. subtilis* NCBI3610 (biofilm) compared to biofilm-deficient *B. subtilis* (Δ*epsH*) at day 5 of adulthood, validated by RT-PCR. To calculate the fold change, the expression level of each gene in *C. elegans* fed *B. subtilis* Δ*epsH* or Δ*tasA* was taken as 1. For RNA-seq, three independent biological replicates were processed (see Materials and Methods). Expression analysis with RT-PCR was performed in three independent biological replicates and the mean value of fold change ± SE is presented.

**Figure 4:**
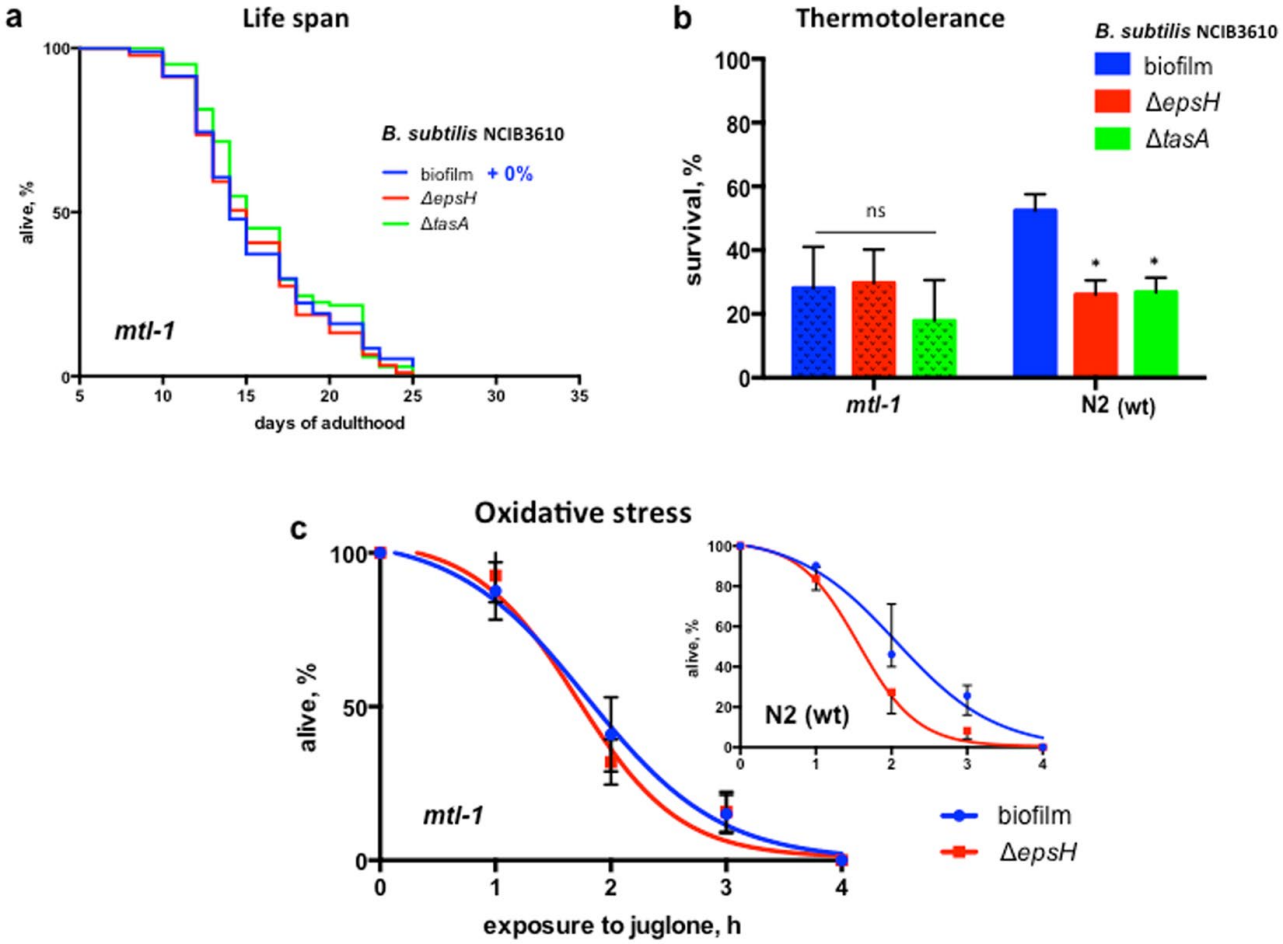
Biofilm acts via induction of metallothionein in *C. elegans*. In each case the average values ± SD from at least three independent experiments are plotted. Each experiment includes at least 60 worms per condition. (**a**) MTL-1 is required for biofilm-dependent lifespan extension. *C. elegans mtl-1* (tm1770) were fed either biofilm-forming *B. subtilis* (biofilm, blue) or its biofilm-deficient derivatives (Δ*epsH* and Δ*tasA*, red and green, respectively). The graph is representative of three independent biological replicates. Median lifespan, days: biofilm–15; Δ*epsH*–15; Δ*tasA*–15. The log-rank test p-value for each experiment is >0.05, Supplementary Table S3. (**b**) MTL-1 is required for biofilm-mediated thermotolerance. Wild-type (N2) and *mtl-1* (tm1770) *C. elegans* were grown in parallel on indicated bacterial strains until day 5 of adulthood and then subjected to heat shock at 33 °C for 3 hours. Surviving animals were scored after 20 hours of recovery at 20 °C. The graph represents mean values ± SD from free independent biological replicates. One-way ANOVA *p*-value = 0.4800. (**c)** MTL-1 is required for biofilm-mediated resistance to oxidative stress. Five-day old wild-type (N2) and *mtl-1* (tm1770) *C. elegans* were grown on indicated bacterial strains, transferred to plates supplemented with juglone (90 μM) and scored for survival every hour. The graph represents mean values ± SD from free independent biological replicates. Also see Supplemental Table S1.

*mtl-1* encodes metallothionein–a small cysteine-rich metal-binding protein that has been implicated in longevity and proteotoxic stress^43^. It is regulated by DAF-16, a FOXO transcription factor broadly involved in lifespan regulation^44, 45^. Indeed, biofilm-producing bacteria failed to extend the lifespan of either *mtl-1* or *daf-16* mutant worms (Fig. 4a and Supplementary Fig. S7a), indicating that MTL-1 plays a primary role in biofilm-mediated longevity.

MTL-1 is known to be upregulated by elevated temperature^46^, although its role in the heat shock response remains unclear. We found that the biofilm-mediated increase in thermotolerance was abrogated in *mtl-1* knockout worms (Fig. 4b), thus implicating MTL-1 in biofilm-mediated heat resistance. Curiously, this effect on thermotolerance was independent of DAF-16 (Supplementary Fig. S7b), suggesting alternative modalities of *mtl-1* regulation in response to the biofilm.

Mammalian metallothioneins function in the oxidative stress response^47^. We, therefore, examined whether the observed upregulation of MTL-1 by biofilm-forming *B. subtilis* improves *C. elegans* oxidative stress resistance. Indeed, *mtl-1* knock-out worms were not protected from juglone by biofilm-forming bacteria (Fig. 4c and Supplementary Table S1).

**Figure 5:**
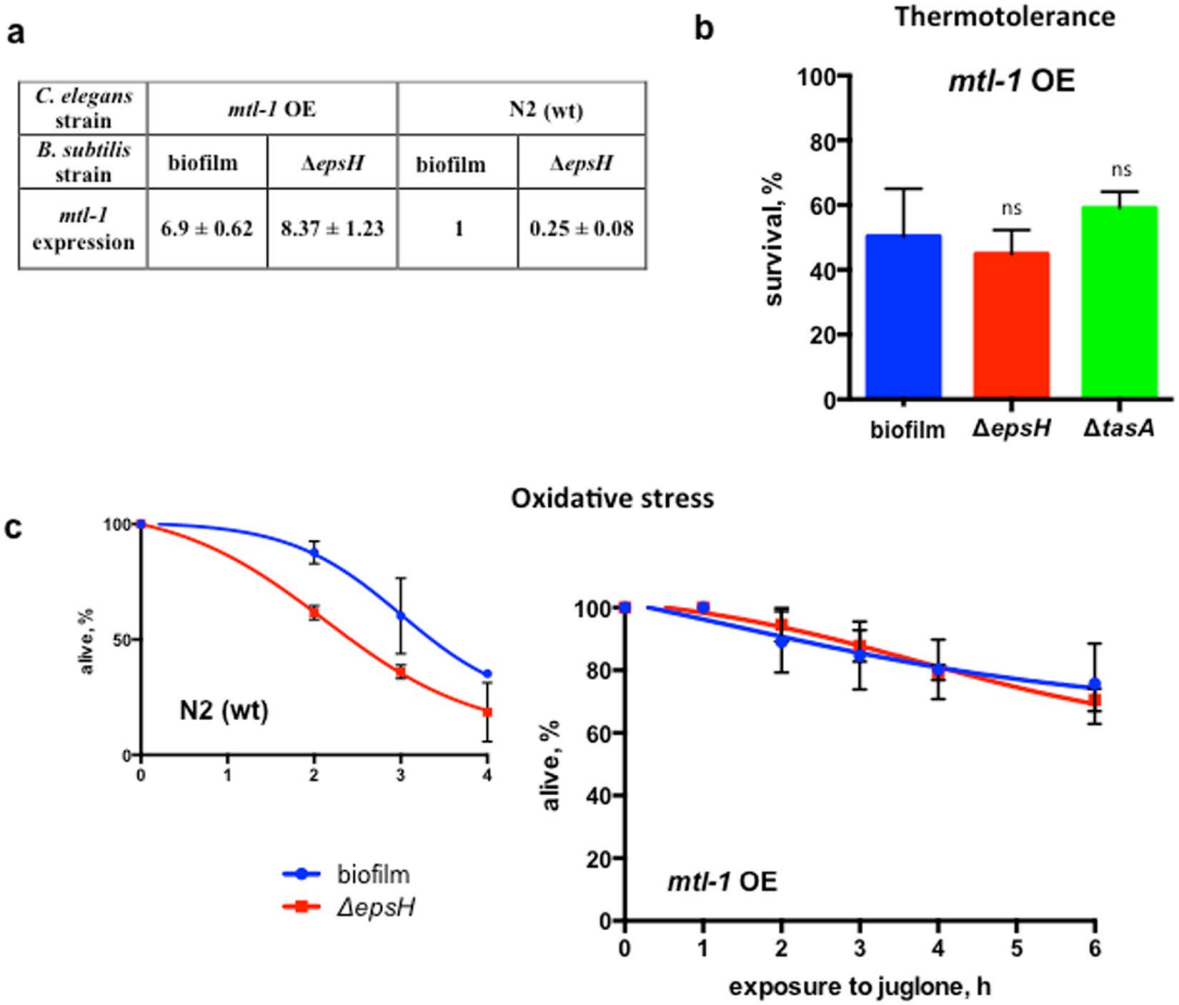
*mtl*-1 overexpression abrogates biofilm-mediated effects. In each case the average values ± SD from at least three independent experiments are plotted. Each experiment includes at least 60 worms per condition. (**a**) Relative levels of *mtl-1* expression. The relative level of *mtl-1* mRNA was calculated as the fold change from the expression level in *C. elegans* N2 fed on wild-type *B. subtilis* (biofilm) using RT-PCR. (**b**) The *B. subtilis* biofilm fails to increase the thermotolerance of *C. elegans* overexpressing *mtl-1*. Five-day old *mtl-1* OE (WU1394) grown on *B. subtilis* NCIB3610 (biofilm) or its biofilm-deficient derivative (Δ*epsH*) were heat shocked at 33 °C for 5 hours. The duration of heat shock was increased from 3 to 5 hours because 100% of *mtl-1* OE worms survived a 3 hour heat-shock treatment regardless of the bacterial strain. Surviving animals were scored after 20 hours of recovery at 20 °C. One-way ANOVA p-value = 0.2877. (**c**) The *B. subtilis* biofilm fails to promote resistance to oxidative stress in worms overexpressing *mtl-1*. Five-day old *mtl-1* OE (WU1394) grown on *B. subtilis* NCBI3610 (biofilm) or its biofilm-deficient derivative (Δ*epsH*) were transferred to plates supplemented with juglone (90 μM) and scored for survival every hour. See Supplementary Table S1.

To further support the hypothesis that MTL-1 plays the principle role in biofilm-mediated effects we studied a *C. elegans* mutant that overexpresses *mtl-1* (*mtl-1* OE) (Fig. 5a). In the background of higher *mtl-1* expression, the *B. subtilis* biofilm failed to promote worm thermotolerance or oxidative stress resistance (Fig. 5b and c).

Taken together, these results demonstrate that upregulated MTL-1 expression accounts for biofilm-associated *C. elegans* phenotypes, including extended lifespan and increased resistance to physical and chemical stress.

### Induction of HSP70 by a commensal biofilm improves C. elegans heat resistance

**Figure 6:**
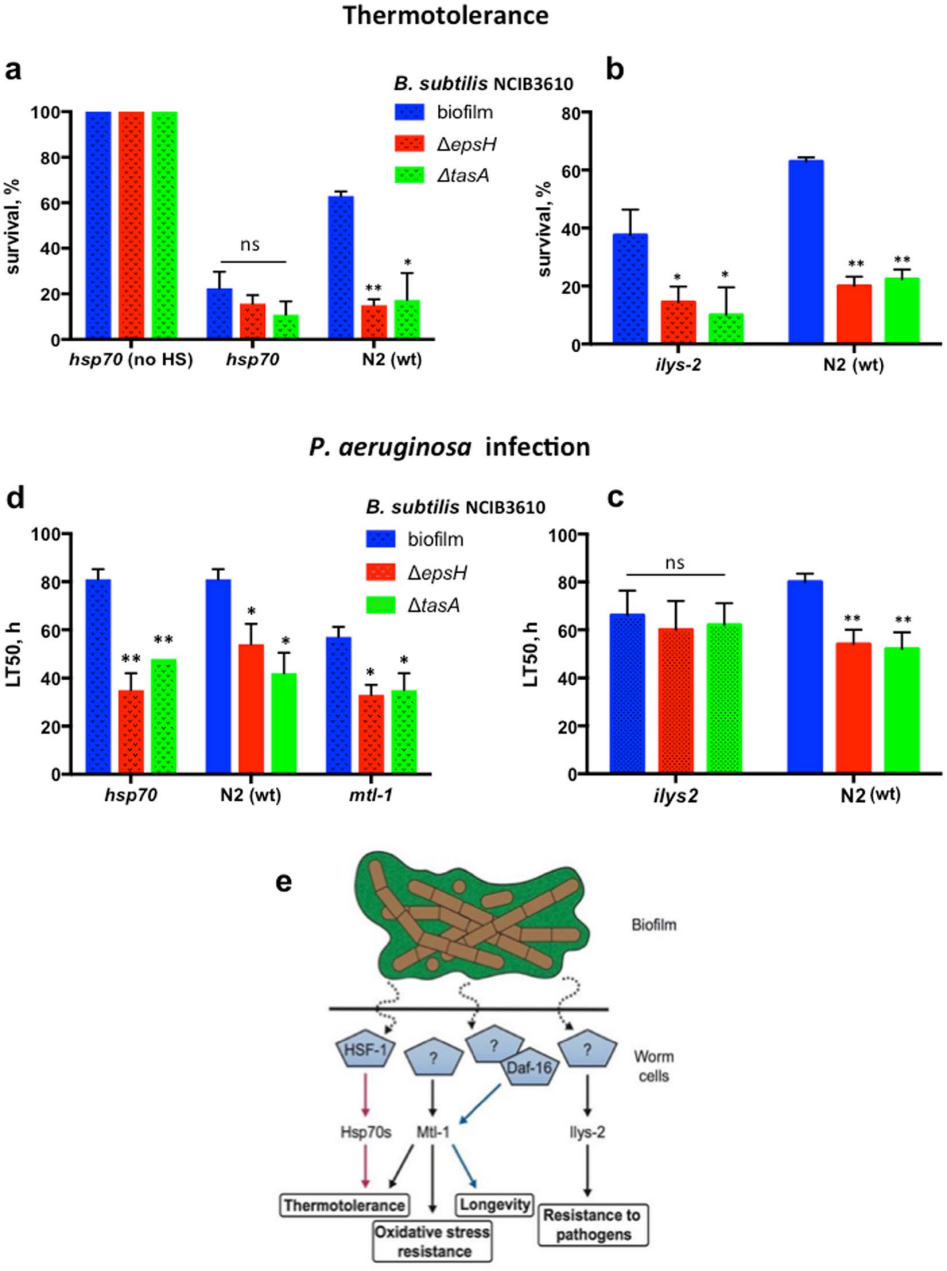
HSP70 and lysozyme contribute to biofilm-mediated resistance to heat and infection. (**a**) *C. elegans hsp-70* (*F44E5.4* and *F44E5.5*) are required for biofilm-induced thermotolerance. Wild-type (N2) and *hsp70*-deficient *C. elegans* were grown in parallel on biofilm-forming (biofilm) and biofilm-deficient (Δ*epsH*, Δ*tasA*) *B. subtilis*. Five-day old worms were subjected to heat shock as in Fig. 1a. Mean values ± SD from three independent biological replicates are plotted. Each biological replicate includes at least 60 worms per condition. One-way ANOVA *p*-value = 0.1210. (**b**) ILYS-2 is dispensable for the biofilm-mediated increase in *C. elegans* thermotolerance. N2 and *ilys-2* worms were grown in parallel on indicated bacterial strains until day 5 of adulthood and then subjected to heat shock at 33 °C for 3 hours. Surviving animals were scored after 20 hours of recovery at 20 °C. (**c**) Biofilm-dependent induction of *ilys-2* is essential for resistance to *P. aeruginosa* infection. Five-day old wild-type (N2) and *ilys2* worms grown in parallel on indicated *B. subtilis* strains were transferred to *P. aeruginosa* PA14 seeded plates and survival was scored every 6 hours. LT50 was taken from Kaplan-Meier survival curves (median survival data). For the log-rank test *p*-values see Supplementary Table S2. (**d**) MTL-1 and HSP70 are not required for biofilm-dependent resistance to *P. aeruginosa* infection. Five-day old wild-type (N2), *mtl-1* (tm1770) and *hsp70* knockout worms grown in parallel on indicated *B. subtilis* strains were transferred to *P. aeruginosa* PA14 seeded plates and survival was scored every 6 hours. LT50 was taken from Kaplan-Meier survival curves (median survival data). For the log-rank test p-values see Supplementary Table S2. (**e**) Biofilm-induced signaling in *C. elegans*. Feeding *C. elegans* biofilm-forming *B. subtilis* induces expression of MTL-1, Hsp70 (F44E5.4 and F44E5.5) and ILYS-2, partly via HSF-1 and DAF-16 activation (blue and red arrows). Pentagons indicate transcription factors. Upregulation of these biofilm responsive genes in host organism results in enhanced lifespan, thermotolerance, and resistance to oxidative stress and pathogenic infection.

*F44E5.4* and *F44E5.5* encode two isoforms of inducible heat shock protein 70 (HSP70), a family of evolutionarily-conserved molecular chaperones that are crucial for survival at elevated temperatures^48^. The *F44E5.4* and *F44E5.5* genes have overlapping promoters from which transcription proceeds in divergent directions^49^. Their transcription is, at least partially, controlled by HSF-1, a master regulator of the heat-shock response^50^ that has been shown to contribute to *C. elegans* lifespan extension^51^. We investigated whether the *B. subtilis* biofilm improves thermotolerance and increases lifespan via HSF-1-dependent activation of heat shock proteins. Thermotolerance and lifespan assays in an *hsf-1* mutant showed that HSF-1 contributes to biofilm-activated resistance to heat-shock, but not to biofilm-induced lifespan extension (Supplementary Fig. S7d and c). We next examined the effect of the biofilm on the thermotolerance of *F44E5.4/F44E5.5* double mutant worms and found that the biofilm was unable to improve their thermotolerance (Fig. 6a). Thus, our results demonstrate that the commensal biofilm activates HSF-1, which in turn leads to induction of specific *hsp70* genes and, as a result, causes increased resistance to elevated temperatures.

### ILYS-2 is required for biofilm-mediated protection against P. aeruginosa infection

As described earlier, the *B. subtilis* biofilm promotes *C. elegans* resistance to *P. aeruginosa* (Fig. 1e). A commensal microbiota is known to stimulate the immune response of higher organisms, thereby promoting their survival during infection^52^. Among the few genes upregulated in response to the biofilm was *ilys-2* (Table 1), which encodes one of the invertebrate lysozymes that function in the *C. elegans* innate immune response^53^. We, therefore, examined whether ILYS-2 contributed to biofilm-mediated resistance against *P. aeruginosa* infectio*n*. We constructed *C. elegans ilys-2* knockout mutant worms and subjected them to the *P. aeruginosa* slow killing assay, as described earlier. In the absence of *ilys-2* the *B. subtilis* biofilm was unable to protect against infection (Fig. 6c, Supplementary Table S2 and Supplementary Fig. S8). Notably, neither MTL-1 nor HSP70s (F44E5.4 and F44E5.5) were required for the biofilm-dependent increase in resistance to *P. aeruginosa* (Fig. 6d, Supplementary Table S2 and Supplementary Fig. S8). Likewise, biofilm was still able to augment thermotolerance of the *ilys-2* knockout (Fig. 6b). Thus, biofilm-dependent induction of *ilys-2* specifically promotes *C. elegans* resistance to pathogenic infection.

## Discussion

Metazoan hosts and their commensal microbiota have coevolved and established intimate relationships for more than 500 million years. Only recently have we begun to understand the complexity of these interactions^7^. Germ-free mice display a plethora of physiological and pathological traits, including decreased digestive activity, body fat, muscle wall thickness, and cytokine and serum immunoglobulin production accompanied by increased susceptibility to infection^54^. Colonization of germ-free mice with a single human symbiotic species, *Bacteroides thetaiotaomicron*, alters the transcriptional response of various tissues, resulting in improved nutrient uptake, metabolic changes, and restoration of the mucosal barrier function^55^. Although it is recognized that the majority of bacteria on the gut epithelium and within the mucus layer exist as biofilms^56^, little is known of the effects of these bacterial populations on the health and disease susceptibility of their hosts.

We utilized a defined model system composed of *B. subtilis* and *C. elegans* to investigate the role of non-pathogenic biofilm-host interaction in host physiology and fitness. Both organisms proliferate in the same ecological niche and potentially represent a natural system that was established long before the evolutionary advent of vertebrates^9, 16^ (Supplementary Discussion). Our data demonstrate that the *B. subtilis* biofilm endows *C. elegans* with important survival advantages, such as increased resistance to heat, oxidants, and pathogenic infection. The biofilm also augments *C. elegans* longevity under undisturbed growth conditions. Notably, these beneficial effects are not due to better colonization of the *C. elegans* host, as we did not detect any biofilm-dependent difference in the lumenal bacterial load (Supplementary Fig. 2). Rather, we found that specific changes in host gene expression, such as induction of the stress (*mtl-1* and *hsp-70*) and innate immune (*ilys-2*) responses, underlie the mechanism of beneficial biofilm-host interactions (Fig. 6e). Lysozymes are important in controlling bacterial population in *C. elegans* intestine^53^ (Fig. 6c and Supplementary Fig. S8). Therefore, *ilys-2* induction may help to curb the accumulation of biofilm-proficient *B. subtilis* in the gut (Supplementary Fig. S2).

Our work demonstrates that the beneficial effects of biofilm on the worm require live bacteria (Fig. 3). Recently, the positive influence of biofilm-specific bacterial metabolites on *C. elegans* was also reported^18^. Notably, in this work *C. elegans* were fed *B. subtilis* spores, which is a poor food source that delays animals development and, thus, potentially induces a caloric restriction response^37^. It was proposed that nitric oxide and a quorum sensing factor are the metabolites responsible for biofilm mediated effects^18^. However, we have demonstrated that *B. subtilis* makes NO and extends *C. elegans* lifespan independently of biofilm formation^13^. Furthermore, the deletion of a biofilm matrix gene (*bslA*) and quorum-sensing factor (*csf*) additively shorten *C. elegans* lifespan^18^, indicating that CSF increases the lifespan independently of biofilm. Further studies are required to find the biofilm-specific bacterial metabolites that are responsible for the observed *C. elegans* phenotypes.

We found that the expression of a small group of *C. elegans* genes was specifically altered by biofilm formation (Table 1, Supplementary Table S5). Both DAF-16 and SKN-1 are important for *C. elegans* oxidative stress resistance^43, 57^. As we did not detect any significant change of SKN-1-dependent genes (Supplementary Table S5) or SKN-1 cellular localization (not shown) in response to biofilm we assumed that *skn-1* is dispensable for biofilm-mediated effects. In contrast, DAF-16-dependent *mtl-1* was upregulated by biofilm (Table 1 and Supplementary Table S5). MTL-1, is a member of the family of metallothioneins, a group of small cysteine-rich proteins with high affinity for metal ions that are widely distributed among species ranging from bacteria to mammals^58^. Metallothioneins function to maintain metal and redox homeostasis and to protect cells against oxidative stress and various genotoxic and proteotoxic agents^58^. In mammals, metallothioneins have also been implicated in neuronal tissue regeneration, modulation of metabolic activity, and suppression of pro-inflammatory and pro-apoptotic responses^59, 60^. The levels of metallothionein are elevated in various tissues of certain strains of long-lived dwarf mice^61^, and the targeted overexpression of metallothionein in cardiac tissue extended the lifespan of wild type mice by 14%^62^. In flies, upregulation of MTL in either motor neurons or the peripheral nervous system was correlated with an approximately 40% increase in lifespan and increased resistance to iron and cadmium toxicity^63^.

*C. elegans* harbors two metallothionein genes (*mtl-1* and *mtl-2*). MTL-1 is constitutively and inducibly expressed, respectively, in the terminal bulb and intestine^64^. MTL-1 is under DAF-16 control^43, 44^, but its role in *C. elegans* longevity remains unclear. We identify MTL-1 as a major determinant of increased *C. elegans* lifespan and stress resistance in response to a commensal biofilm (Figs 4 and 5). DAF-16 is required for biofilm-dependent lifespan extension, but is dispensable for augmented thermotolerance (Supplementary Fig. S7a and b), suggesting that other transcription factors stimulate the biofilm-stimulated expression of *mtl-1* to render *C. elegans* resistant to heat stress (Fig. 6e). Further studies will address the molecular details of biofilm-mediated regulation of DAF-16, and potential tissue-specificity of biofilm effects.

Evolutionarily conserved HSP70 proteins also contribute to the biofilm-mediated increase in *C. elegans* thermotolerance (Fig. 6a). We show that two HSP70 encoding genes, *F44E5.4* and *F44E5.5* that are transcribed from overlapping divergent promoters are induced by the *B. subtilis* biofilm (Table 1). The HSP70 family of chaperones performs a multitude of cytoprotective functions in all organisms^48^. The *C. elegans* genome contains six full-length cytosolic HSP70-encoding genes^49^. It was shown that the expression of *F44E5.4* and *F44E5.5* increases upon heat shock^65^, hypoxic stress^66^, and pathogen infection^67^. The products of these genes have not been well studied and their specific role in *C. elegans* remains unknown. Our data implicate these HSP70 paralogs in thermotolerance, which is controlled by the commensal biofilm via HSF1 (Fig. 6a and Supplementary Fig. S7c). Interestingly, Donato *et al*. reported that HSF-1 is required for biofilm mediated life span extension, while our results show that HSF-1 is only required for biofilm-mediated thermotolerance (Supplementary Fig. S7c and d). It has been shown that HSF-1 is involved in a dietary restriction response^68^. *B. subtilis* spores may trigger the dietary restriction response because they are a poor nutritional source^37^. This may explain the apparent discrepancy between our results and those reported by Donato *et al*., who fed worms on bacterial spores.

Natural microbiota primes and balances the host immune system^52^. Activation of the innate response by commensal bacteria leads to accumulation of diverse types of antimicrobial factors, including lactic acid and bacteriocins, in the gastrointestinal tract to restrain the bacterial load^54^. The invertebrate lysozyme, ILYS-2, functions in the *C. elegans* innate immune system^53^. It was shown to be upregulated during infection, suggesting its role in organismal defense against pathogens. However, whether ILYS-2 actually contributes to the animals’ survival upon infection has not been determined. Our data show that the *B. subtilis* biofilm specifically stimulates *ilys-2* expression, increasing the resistance of *C. elegans* to killing by *Pseudomonas* (Fig. 6c and e and Table 1).

In this work we focus on the overall response of the host to non-pathogenic biofilm, leaving the identification of inter-species and inter-tissue signaling for future studies. The beneficial effects of bacterial biofilm can be mediated by *C. elegans* intestinal cells and by non-autonomous cell signaling. Although the expression patterns of *mtl-1* and *ilys-2* in the intestinal cells have been characterized, little is known about *F44E5.4* and *F44E5.5* ^44, 53^. Overall, considering the high structural and functional conservation of metallothioneins, HSP70, and lysozymes throughout evolution, and the ancient origin of the regulatory networks with which they are associated (Fig. 6e), it is tempting to hypothesize that biofilms formed by the mammalian microbiota also confer beneficial effects on their host via similar regulatory pathways.

## Matherials and Methods

### C. elegans strains and growth conditions

Wild-type *C. elegans* (N2), PS3551 (*hsf-1(sy441)* I), CF1038 (*daf-16(mu86) I*), DA1116 (*eat-2* (ad1116) II) strains were obtained from the *Caenorhabditis* Genetics Center. The *C. elegans mtl-1* knock-out (tm1770) strain was obtained from National Bioresource Project for the Experimental Animal “Nematode *C. elegans*” (Tokyo, Japan) and outcrossed to *C. elegans* N2 6 times before use in experiments. The *C. elegans mtl-1* overexpression strain WU1394 (pKD8 amEx183(Pmtl-1(WT)::MTL-1::GFP::mtl-1 3’UTR; myo-3::mCherry) was kindly provided by Kerry Kornfeld, Washington University in St. Louis^69^. *C. elegans ilys-2* and *hsp70* knock-out mutants were constructed in this work (Supplementary Table S6). Nematodes were handled according to standard method^70^. Worms were grown on NGM (Research Product International Corp.) at 20 °C, unless otherwise indicated, and routinely maintained on *E. coli* OP50. To transfer the nematodes to *B. subtilis* strains eggs were purified by the alkaline hypochlorite method^71^. In all assays animals were grown on the indicated *B. subtilis* strains for at least one, but not more than three, generations prior to use in experiments.

### Bacterial strains and growth conditions

Wild-type undomesticated *B. subtilis* NCIB3610, CYBS-5 and their biofilm-deficient derivatives Δ*epsH*::tet (DS76), Δ*tasA*::spec (SSB505), the Δ*tasA* complemented strain (Δ*tasA*::spec, *amyE*::P*yqxM-yqxM-sipW-tasA* (FC202)), CYBS-5 Δ*epsH*::tet and *B. subtilis sacA*::PtasA-mKate2 (TMN503) were isolated or constructed previously^17, 72–74^. *B. subtilis* PtasA-mKate2 ∆*sinI* (ASD390) was constructed in this study by transducing TMN1075 with phage from TMN503. TMN1075 is a markerless ∆*sinI* strain which was constructed using pTMN994^74^. *P. aeruginosa* PA14 was kindly provided by Fred Ausubel^26^. *Pseudomonas fluorescens* Pf0-1 and its Δ*lapA* derivative were the generous gift of George A. O’Toole, Geisel School of Medicine at Dartmouth. *L. rhamnosus* GG (ATCC53103) and its Δ*spaCBA* (CMPG5357) counterpart were kindly provided by Sarah Leeber, University of Antwerp^25^. *E. coli* OP50 was obtained from the *Caenorhabditis* Genetics Center (Supplementary Table S6).

Overnight bacterial cultures were grown in LB media at 30 °C with agitation and 50 µl were spread atop NGM agar plates. Seeded plates were incubated at 25 °C for ~20 hours and then for 2 hours at 20 °C before worms were transferred onto them.

For the experiments with antibiotic-treated agar plates, overnight *B. subtilis* lawns on NGM plates were treated with a mixture of antibiotics (500 μg/ml carbenicillin and 100 μg/ml kanamycin in final concentration) and dried for 1.5 hours at room temperature. Plates were shifted to 20 °C for 2 hours before larval stage L4 worms were transferred onto them.

Worms were transferred to freshly treated plates every other day.

For experiments with *L. rhamnosus* bacterial cultures were grown in MRS medium at 30 °C without agitation and 150 µl were spread atop NGM agar plates. The plates were equilibrated at 20 °C for 2 hours and used immediately.

### Lifespan analysis

Lifespans were monitored at 20 °C without FUDR as described previously^75^ with the following changes. Previously age –synchronized L4 nematodes were transferred to fresh agar plates of the same bacterial strain and the following day was counted as day one in all lifespan measurements. Nematodes were judged as dead when they ceased pharyngeal pumping and did not respond to prodding with a platinum wire. Escaped animals or animals with internal hatching were not included in the lifespan calculations. Kaplan-Meier survival curves were generated using the GraphPad Prism6 statistical analysis software package. Kaplan-Meier curves were compared with the Log-rank (Mantel-Cox) statistical test.

### Thermotolerance assay

Thermotolerance assays were performed as described previously for the single-point survival assay^76^ with the following changes: previously age–synchronized L4 animals were transferred to fresh agar plates of the same bacterial strain and subsequently transferred every other day. Day-5-adults were subjected to heat shock in a water bath at 33 °C (unless otherwise indicated) for the indicated time. After heat shock, worms were shifted to an air incubator at 20 °C and surviving animals were scored after an approximate 20 hour recovery (unless otherwise indicated).

For the experiments with *L. rhamnosus* GG (ATCC53103) animals were grown on *E. coli* OP50 until L4 stage, since *Lactobacillus* do not support *C. elegans* development. L4-staged worms were treated 3 times with the mix of antibiotics (4 mg/ml carbenicillin and 0.8 mg/ml kanamycin) and then transferred to either *L. rhamnosus* GG or its biofilm-deficient derivative (Δ*spaCBA*). Consequently animals were transferred to the fresh plates with corresponding bacterial strains every day until day 5 of adulthood and plates were monitored for potential *E. coli* OP50 contamination. Worms were subjected to the heat-shock as described earlier.

### Oxidative stress resistance assay

Oxidative stress resistance was determined as described^77^ with the following changes: Fresh 2 mg/ml juglone solution was prepared by dissolving juglone in ethanol (200 proof for molecular biology, Sigma) for 30 min. We used a motorized pestle to facilitate the dilution process. Agar plates containing juglone (90 μM) were freshly prepared before each experiment using fresh juglone solution and were dried in the sterile hood for 1 hour. Worms were age-synchronized as described earlier and transferred to fresh plates with the indicated *B. subtilis* strain every other day. Day 5 adult animals were transferred to juglone containing plates without bacteria and surviving animals were counted every hour. The percentages of surviving animals was fit to a sigmoidal curve and the LT50 was determined and analyzed for statistical difference using the GraphPad Prism6 statistical analysis software package.

### P. aeruginosa slow killing assay

The *P. aeruginosa* slow killing assay was performed essentially as described in ref. 26. *C. elegans* were age-synchronized and grown until day 5 of adulthood as described earlier. An overnight culture of *P. aeruginosa* PA14 in BHI broth was spread on modified NGM (0.35% peptone) agar plates, incubated at 37 °C for 24 hours and then at 25 °C for 18 hours. 30–50 five-day old adult worms were transferred to the *P. aeruginosa* PA14 plates and examined for survival at 25 °C every 6 hours.

### Plasmid construction

An *ilys-2* homology repair template, pBADilys2, was designed to delete the first three exons of *ilys-2*. Two 1-kb homologous regions were PCR-amplified from *C. elegans* N2 genomic DNA (for primer sequences see Supplementary Table S7).

The *Unc-119*(+) rescue construct was amplified from punc-119cbr (Addgene #32568). The resulting DNA fragments were gel-purified and assembled into the pBAD cloning vector using the Gibson assembly kit (NEB).

The *Hsp-70* homology repair template pBADhsp70 was designed to delete the common promoter region of F44E5.4 and F44E5.5. Using *C. elegans* N2 genomic DNA as a template, two homologous regions (1.81-kb and 1.877) were PCR-amplified (for primer sequences see Supplementary Table S6).

The *Unc-119*(+) rescue construct was amplified from punc-119cbr (Addgene #32568). The resulting DNA fragments were gel-purified and assembled into the pBAD cloning vector using the Gibson assembly kit (NEB).

sgRNA encoding vectors: pSgRilys2 and pSgRhsp70. sgRNA sequences were designed using the Benchling Genome Engineering software and cloned into pDD162 (Addgene #47549) with forward primer 5′-N_19_GTTTTAGAGCTAGAAATAGCAAGT-3′ and reverse primer 5′-CAAGACATCTCGCAATAGG-3, where:

N19 pSgRilys2 = atataagccgtcaaggtag,

N19 pSgRhsp70 = gcatcaaatactgtattctc

### ilys-2 and hsp-70 mutants construction

*ilys-2* and *hsp70* mutants were constructed using CRISPR-Cas9 essentially as described in ref. 78. Briefly, *C. elegans* HT1593 were injected with a plasmid mixture containing a homology repair template (10 ng/μl), Cas9-a (50 ng/μl), and co-injection markers. Injections were performed by Knudra Transgenics (http://www.knudra.com/). Animals with an unc-119 rescued phenotype that do not carry the co-injection markers were selected at 20 °C and subjected to PCR-genotyping (for primer sequences see Supplementary Table S7). Resulting mutant strains were outcrossed 6 times to wild-type N2.

### RNA isolation, next-generation sequencing, and differential expression analysis

Approximately 200 five-day-old adult worms grown on wild-type or ∆*epsH B. subtilis* were collected and washed in S-buffer, and total RNA was isolated as described in ref. 79. A TrueSeq RNA Sample Preparation Kit v2 (Illumina) was used to prepare cDNA libraries for RNA-Seq from 1 μg of total RNA. Three independent biological replicates were used for each experimental condition. The reference genome and annotation data for *C. elegans* (Ensembl assembly based on WS220 build) were downloaded from the Illumina websitm (ftp://igenome:G3nom3s4u@ussd-ftp.illumina.com/Caenorhabditis_elegans/Ensembl/WS220/Caenorhabditis_elegans_Ensembl_WS220.tar.gz). To estimate the expression level of transcripts and test for differential expression between different experimental conditions, the Tophat/Cufflinks/Cuffdiff pipeline was used^80^. Briefly, the RNA-seq reads were trimmed of adaptor sequences and then mapped to the *C. elegans* transcriptome with the Tophat software package^81^ using the bowild-typeie2 aligner and default parameters. The transcripts were assembled and their abundances estimated using the Cufflinks package^82^. A statistical test for differential gene expression was performed using the Cuffdiff tool in the Cufflinks package with a q value (p value adjusted for multiple testing^83^) threshold of 0.05. Analyses of the differential expression data were performed with the R software package (version 2.15.1) using the cummeRbund library (version 2.0).

### Reverse transcription and real-time PCR

Total RNA from 100 worms grown on indicated bacterial strains was extracted as described earlier. Total RNA samples were treated with turbo DNAse (Ambion), and total RNA concentration was measured using NanoDrop1000 (Thermoscientific). Equal amounts of total RNA were reverse transcribed with random primers using SuperScriptII (Invitrogen). 1 μl of the resulting cDNA solution was used for RT-PCR with specific primers (Supplementary Table S6) and Power SYBR Green PCR master mix 2 × (Applied Biosystems) for 40 cycles in a 7300 real-time PCR system (Applied Biosystems) according to the manufacturer’s instructions. The fold change of mRNA levels of each target gene was normalized to the geometric mean of fold change of control genes (*pmp-3* and *cdc-42*).

### Self-brood size and rate of egg production

Experiments were performed essentially as described^84^. Eggs from strain N2 nematodes were isolated, treated with hypochlorite, and incubated for 20 hours at 20 °C in S-buffer without cholesterol^85^. A synchronized population of L1 arrested worms was then placed on NGM agar plates seeded with wild-type or Δ*epsH B. subtilis*. Five stage L4 animals were picked manually and transferred to a new plate. Worms were then transferred twice a day to prevent overcrowding until egg-laying ceased. The progeny were counted 3 days after removal of the parents.

### Post-embryonic development

Experiments were performed essentially as described in ref. 84. Worms were grown on NGM agar plates seeded with wild-type or Δ*epsH B. subtilis*. Unstaged eggs were placed at 20 °C and allowed to hatch for 4 hours. Larvae that hatched during that period were placed singly on fresh agar plates and monitored every 5 hours until they began to lay eggs.

### Fluorescent microscopy and WGA-FITC staining

To fluorescently label exopolysaccharides we used FITC-conjugated wheat germ agglutinin, a lectin from *Triticum vulgaris* (Sigma). The labeling was performed as following: *C. elegans* were grown on *B. subtilis* strains until day 5 of adulthood and then incubated in 50 μl of WGA-FITC solution in PBS (1 μg/ml) for 5 h with agitation.

For microscopic visualization of mKate2 expression and WGA-FITC labeled exopolysaccharides worms were anesthetized with a drop of 5 mM levamisole. Images of 15 worms for each variant were captured at a fixed exposure time using a Zeiss AxioZoom.V16 equipped for fluorescence illumination. Fluorescence intensity was quantified using ImageJ software.

### Motility (thrashing) assay

*C. elegans* were grown on the corresponding *B. subtilis* strains as described previously and trashing rate was measured as in (http://www.phage.dk/plugins/download/wrMTrck.pdf) with the following modifications. At day 2 and day 8 of adulthood 10 worms were placed into the 20 μl drop of M9 buffer on the surface of NGM plate without bacteria and left for 1 min to calibrate. 1 minute videos were taken using Zeiss AxioZoom.V16 equipped with AxioCam HRc camera and body bends per second per worm were calculated using ImageJ software and wrMTrck plugin^86^.

### Colonization assays

The colonization assay was adapted from^87^. 10 worms were picked and placed in a drop of M9 buffer and subsequently washed in 3 drops of M9, the last of which contained 4 mM levamisole for paralysis. Nematodes were then collected and washed in M9 with 1% Triton X-100 and twice with M9 buffer. An aliquot of the final M9 buffer wash was plated as a control for the presence of non-intestinal bacteria. Worms were mechanically disrupted using a motorized pestle. The resulting worm homogenates were serially diluted and plated on LB agar. To calculate the percentage of spores, aliquots of worm homogenate were incubated at 80 °C for 20 min and plated on LB agar. The number of resulting colony forming units (CFU) on LB agar plates was counted after 20 hours at 37 °C. The number of vegetative bacterial cells per worm was calculated by subtracting the CFUs of the non-intestinal control the intestinal spore count from the total number of CFUs and divided by the number of worms per sample.

Alternatively, we used a “guillotine method”, adapted from^88^ which eliminates bacteria stuck in the pharynges, to determine bacterial colonization. Worms were collected and washed as described for the colonization assay and the pharynx was dissected under the microscope. Worms were collected individually in 50 μl of M9 buffer and mechanically disrupted using a motorized pestle. Aliquots of the homogenates of individual worms were plated on LB agar plates. The aliquots were also plated after having been heated at 80 °C for 20 min to evaluate the percentage of spores. The number of vegetative bacterial cells per worm was calculated as described earlier.

### Pumping rate measurements

Pumping rate was measured as the rate of pharyngeal terminal bulb contractions as described (http://www.wormbook.org/chapters/www_measurepharyngeal/measurepharyngeal.html). Briefly, 10 day-one adult worms grown on indicated bacterial strains were examined for 10 seconds, and the number of terminal bulb contractions were counted.

### Worm size measurement

The size of *C. elegans* on *B. subtilis* plates was measure as described in ref. 89. Briefly, the images of the worms grown on bacterial plates were taken at L4 larval stage and day 1, 3, 4 of adulthood using Zeiss AxioZoom.V16 with the set magnification. Next, surface area of individual worms was determined by means of ImageJ software and WormSizer plugin. The recognition of each individual worm by software was reevaluated by eye.

### Transmission Electron Microscopy

5 to 8 living worms were put into the center of 100 µm deep planchette hats that is filled with yeast paste. The hats were coated with hexadecane, sealed in the planchette holder and high pressure freezing commenced with a Wohlwend Compact HPF-01 High Pressure Freezer (Bal-Tec AG, Liechtenstein). The frozen hats were transferred into a mixture of 2% osmium tetroxide, 0.1% uranyl acetate and 2% ddH2O in acetone at liquid nitrogen, and freeze freeze substitution were using Leica EM AFS2 unit. The samples were left in the –90 °C for 96 hours, raised 5 °C per hour to –60 °C and incubated for 12 hours, then to –30 °C for an additional 12 hours, and finally to a temperature of 0 °C for 4 hrs. Three times 1 hour exchanges of pure acetone were used to rinse out the osmium at 0 °C. Infiltration at room temperature began with a mixture of acetone and Embed 812 (Electron Microscopy Sciences, Hatfield, PA) at 1 to 1 for 1 hour, and 1 to 2 overnight. The samples were allowed to sit in pure resin for 4 hours before embedding. The worms were flat embedded with Aclar embedding film and polymerized at 60 °C. Serial semi-thin sections were cut (UC6 microtome; Leica Microsystems) at 1 mm and stained with 1% toluidine blue to evaluate the quality of preservation and find the area of interest. 60 nm ultrathin sections were cut and stained with uranyl acetate and lead citrate by standard methods. Stained grids were examined under Philips CM-12 electron microscope and photographed with a Gatan (4k × 2.7 k) digital camera.

### Data availability

The datasets generated during and/or analysed during the current study are available from the corresponding author on reasonable request.

## Electronic supplementary material

Supplementary Information
