# Supplementary material for: Mechanism of biofilm-mediated stress resistance and lifespan extension in C. elegans
Source: Sci Rep. 2017 Aug 2;7:7137. doi: 10.1038/s41598-017-07222-8 (PMC5540977; doi:10.1038/s41598-017-07222-8)
Supplement: Supplementary file 1 — Supplementary Information [file 41598_2017_7222_MOESM1_ESM.doc]

**Mechanism of biofilm-mediated stress resistance and lifespan extension in *C. elegans***

**Olga Smolentseva^1^, Ivan Gusarov^1^, Laurent Gautier^1^, Ilya Shamovsky^1^, Alicia S. DeFrancesco^2^, Richard Losick^2^, and Evgeny Nudler^1,3^**

**SUPPLEMENTARY INFORMATION**

**Supplementary Discussion**

*B. subtilis - C. elegans pair as a model to study the effects of non-pathogenic biofilms.*

Several recent reports described bacterial species from *C. elegans* in their natural habitat. Even as a minor component *Bacillus* sp. were found among natural bacterial community that populates *C. elegans* gut^1-4^. The bacterial species found by different groups in abundance in *C. elegans* microbiome, such as *Ochrobactrum, Xantomonas, Sphingomonas*, *Pseudomonas, Stenotrophomonas, Leuconostoc and Lactococcus,* also form biofilms^5-9^, however the lack of developed genetics and well-studied mechanisms of biofilm formation precluded us from analyzing the impact of biofilms of those species on *C. elegans* physiology. Moreover, many representatives of bacterial genera identified as an abundant component of worms’ microbiota, such as *Xantomonas* and *Pseudomonas* could be detrimental for *C. elegans*^4,10,11^. On the contrary, the advantages of *B. subtilis* diet for *C. elegans* have been shown previously^12-14^. Considering that *B. subtilis* is the exceptionally well-studied model organism with a defined mechanism of biofilm-formation, it was a natural choice to study the effect of non-pathogenic bacterial biofilm on its host.

*Extracellular matrix per se does not transduce biofilm-mediated phenotypes.*

Our experiments with antibiotics were designed in a way that all the pre-synthesized components of biofilm would still be present on the plates. Prior to the treatment with antibiotics, bacterial plates were grown essentially the same way as in other experiments. After treatment with antibiotics, bacterial cells remain “outside” of the worms, but no vegetative cells are retained “inside” the animal. Since antibiotic treatments abrogated all the beneficial effects of the biofilm (Fig. 1b and Fig. 3d), we concluded that it is the intestinal biofilm that influences the worm physiology.

*Significance of biofilm-mediated phenotypes.*

We altered only one specific property of host microbiota (biofilm), and yet observed significant effect on lifespan (19%) and even more so on stress resistance. Our work describes the effect of biofilm (*exogenous*) mutations on *C. elegans* physiology, which should be compared with the effect of (*exogenous*) probiotics or drugs on lifespan rather than (*endogenous*) mutations in *C. elegans* itself. All effects imposed by the biofilm on lifespan are statistically significant and their magnitude is comparable to that reported in other studies on bacteria-*C. elegans* interactions^13,15,16^.

We also would like to note that both RNA-seq and qRT-PCR were performed using total RNA from whole animals, whereas upregulation of target genes may occur only in a specific tissue(s). Thus, *mtl-1* is differentially expressed in various tissues and is under unique transcriptional control^17^.

**Supplementary References**

1 Niu, Q. *et al.* Changes in intestinal microflora of Caenorhabditis elegans following Bacillus nematocida B16 infection. *Scientific reports* **6**, 20178, doi:10.1038/srep20178 (2016).

2 Baquiran, J. P. *et al.* Culture-independent investigation of the microbiome associated with the nematode Acrobeloides maximus. *PloS one* **8**, e67425, doi:10.1371/journal.pone.0067425 (2013).

3 Montalvo-Katz, S., Huang, H., Appel, M. D., Berg, M. & Shapira, M. Association with soil bacteria enhances p38-dependent infection resistance in Caenorhabditis elegans. *Infection and immunity* **81**, 514-520, doi:10.1128/IAI.00653-12 (2013).

4 Samuel, B. S., Rowedder, H., Braendle, C., Felix, M. A. & Ruvkun, G. Caenorhabditis elegans responses to bacteria from its natural habitats. *Proceedings of the National Academy of Sciences of the United States of America* **113**, E3941-3949, doi:10.1073/pnas.1607183113 (2016).

5 Gu, J. D., Roman, M., Esselman, T. & Mitchell, R. The role of microbial biofilms in deterioration of space station candidate materials. *International biodeterioration & biodegradation* **41**, 25-33 (1998).

6 Venugopalan, V. P. *et al.* Architecture of a nascent Sphingomonas sp. biofilm under varied hydrodynamic conditions. *Applied and environmental microbiology* **71**, 2677-2686, doi:10.1128/AEM.71.5.2677-2686.2005 (2005).

7 Mercier, C. *et al.* Positive role of peptidoglycan breaks in lactococcal biofilm formation. *Molecular microbiology* **46**, 235-243 (2002).

8 Di Bonaventura, G., Spedicato, I., D'Antonio, D., Robuffo, I. & Piccolomini, R. Biofilm formation by Stenotrophomonas maltophilia: modulation by quinolones, trimethoprim-sulfamethoxazole, and ceftazidime. *Antimicrobial agents and chemotherapy* **48**, 151-160 (2004).

9 Leathers, T. D. & Bischoff, K. M. Biofilm formation by strains of Leuconostoc citreum and L. mesenteroides. *Biotechnology letters* **33**, 517-523, doi:10.1007/s10529-010-0450-2 (2011).

10 Dirksen, P. *et al.* The native microbiome of the nematode Caenorhabditis elegans: gateway to a new host-microbiome model. *BMC biology* **14**, 38, doi:10.1186/s12915-016-0258-1 (2016).

11 Berg, M. *et al.* Assembly of the Caenorhabditis elegans gut microbiota from diverse soil microbial environments. *The ISME journal*, doi:10.1038/ismej.2015.253 (2016).

12 Garsin, D. A. *et al.* Long-lived C. elegans daf-2 mutants are resistant to bacterial pathogens. *Science* **300**, 1921, doi:10.1126/science.1080147 (2003).

13 Gusarov, I. *et al.* Bacterial nitric oxide extends the lifespan of C. elegans. *Cell* **152**, 818-830, doi:10.1016/j.cell.2012.12.043 (2013).

14 Iatsenko, I., Yim, J. J., Schroeder, F. C. & Sommer, R. J. B. subtilis GS67 protects C. elegans from Gram-positive pathogens via fengycin-mediated microbial antagonism. *Current biology : CB* **24**, 2720-2727, doi:10.1016/j.cub.2014.09.055 (2014).

15 MacNeil, L. T., Watson, E., Arda, H. E., Zhu, L. J. & Walhout, A. J. Diet-induced developmental acceleration independent of TOR and insulin in C. elegans. *Cell* **153**, 240-252, doi:10.1016/j.cell.2013.02.049 (2013).

16 Brooks, K. K., Liang, B. & Watts, J. L. The influence of bacterial diet on fat storage in C. elegans. *PloS one* **4**, e7545, doi:10.1371/journal.pone.0007545 (2009).

17 Moilanen, L. H., Fukushige, T. & Freedman, J. H. Regulation of metallothionein gene transcription. Identification of upstream regulatory elements and transcription factors responsible for cell-specific expression of the metallothionein genes from Caenorhabditis elegans. *The Journal of biological chemistry* **274**, 29655-29665 (1999).

**Supplementary Tables**

**Supplementary Table S1: Summary of oxidative stress assays.**

| Set # | *C. elegans* strain | *B. subtilis* strain | LT50, h ±SE | N | *p-*value | Significance |
| --- | --- | --- | --- | --- | --- | --- |
| 1 | N2 | wt | 2.406 ±0.06 | 154 | <0.0001 | **** |
|  |  | Δ*epsH* | 1.29 ±0.11 | 211 |  |  |
| 2 | *mtl-1* | wt | 1.797 ±0.08 | 256 | 0.3985 | ns |
|  |  | Δ*epsH* | 1.705 ±0.07 | 230 |  |  |
|  | N2 | wt | 2.067 ±0.10 | 205 | <0.0001 | **** |
|  |  | Δ*epsH* | 1.562 ±0.04 | 199 |  |  |
| 3 | *mtl-1* OE | wt | N/A | 146 | 0.4433* | ns |
|  |  | Δ*epsH* | N/A | 156 |  |  |
|  | N2 | wt | 3.006 ±0.19 | 116 | 0.0043 | ** |
|  |  | Δ*epsH* | 2.124 ±0.24 | 100 |  |  |

* - measured by multiple t-test per time point.

Experimental and control analyses, which were performed side-by-side, are indicated by the same set number (1, 2 etc.) in the first column. T-test p-values (GraphPad Prism6) were calculated with respect to the same strain of *C. elegans* grown on wild-type *B. subtilis* in the same experiment.

**Supplementary Table S2: Summary of all *P. aeruginosa*** killing assays.

| **Experiment #** | ***C. elegans* strain** | ***B. subtilis* strain** | **Median survival time, h** | **Log-rank P-value** | **Significance** | **died/total** |
| --- | --- | --- | --- | --- | --- | --- |
| 1 | N2 (wt) | wild-type (biofilm) | 86 |  |  | 53/74 |
| 2 |  |  | 72 |  |  | 38/58 |
| 3 |  |  | 84 |  |  | 33/63 |
| 5 |  |  | 78 |  |  | 40/66 |
| 6 |  |  | 84 |  |  | 32/59 |
| 7 |  |  | 78 |  |  | 42/68 |
| 1 |  | Δ*epsH* | 54 | 0.0397 | * | 53/62 |
| 2 |  |  | 54 | 0.0049 | ** | 48/59 |
| 3 |  |  | 54 | 0.0024 | ** | 40/58 |
| 5 |  |  | 54 | 0.0094 | ** | 39/74 |
| 6 |  |  | 60 | 0.0001 | *** | 43/51 |
| 7 |  |  | 48 | 0.0035 | ** | 34/63 |
| 2 |  | Δ*tasA* | 60 | 0.0049 | ** | 53/55 |
| 3 |  |  | 48 | 0.0024 | ** | 51/66 |
| 5 |  |  | 60 | 0.0094 | ** | 44/60 |
| 6 |  |  | 48 | 0.0001 | *** | 53/76 |
| 7 |  |  | 36 | 0.0035 | ** | 43/73 |
|  | | | | | | |
| 3 | *ilys-2* | wild-type (biofilm) | 72 |  |  | 37/75 |
| 4 |  |  | 72 |  |  | 29/56 |
| 5 |  |  | 54 |  |  | 43/67 |
| 3 |  | Δ*epsH* | 48 | 0.0846 | ns | 42/69 |
| 4 |  |  | 72 | 0.7149 | ns | 36/65 |
| 5 |  |  | 60 | 0.4637 | ns | 40/62 |
| 3 |  | Δ*tasA* | 54 | 0.0846 | ns | 47/87 |
| 4 |  |  | 72 | 0.7149 | ns | 38/74 |
| 5 |  |  | 60 | 0.4637 | ns | 44/66 |
|  | | | | | | |
| 6 | *hsp-70* | wild-type (biofilm) | 78 |  |  | 42/68 |
| 7 |  |  | 84 |  |  | 38/70 |
| 6 |  | Δ*epsH* | 40 | 0.0059 | ** | 34/63 |
| 7 |  |  | 30 | < 0.0001 | **** | 36/59 |
| 6 |  | Δ*tasA* | 48 | 0.0059 | ** | 43/70 |
| 7 |  |  | 48 | < 0.0001 | **** | 44/70 |
|  | | | | | | |
| 6 | *mtl-1* | wild-type (biofilm) | 54 |  |  | 26/54 |
| 7 |  |  | 60 |  |  | 48/67 |
| 6 |  | Δ*epsH* | 30 | 0.0115 | * | 46/58 |
| 7 |  |  | 36 | 0.0085 | ** | 45/63 |
| 6 |  | Δ*tasA* | 30 | 0.0115 | * | 38/55 |
| 7 |  |  | 40 | 0.0085 | ** | 37/61 |

Each data set (experiment #) was analyzed by building Kaplan-Meier survival curves and median survival time calculated. Independent experimental and control analyses, which were performed side-by-side, are indicated by the same number (1, 2 etc.) in the first column. The log-rank test p-values (GraphPad Prism6) were calculated with respect to the same strain of *C. elegans* grown on wild-type *B. subtilis* in the same experiment.

**Supplementary Table S3: Summary of aging experiments.**

| **Experiment #** | ***C. elegans* strain** | **Bacterial strain** | **Median survival, days** | **Log-rank p-value** | **Significance** | **died**  **/total** | **Average ± SEM** | **Average % decrease**  **vs**  ***B. subtilis* wt** |
| --- | --- | --- | --- | --- | --- | --- | --- | --- |
| 1 | N2 | *E. coli*  OP50 | 14 | < 0.0001 | **** | 70/85 | 13.5  ± 0.5 |  |
| 5 |  |  | 13 | < 0.0001 | **** | 67/75 |  |  |
|  |  |  |  |  |  |  |  |  |
| 1 | N2 | *B. subtilis*  wild-type | 17 |  |  | 61/70 | 17.6  ± 0.4 |  |
| 2 |  |  | 19 |  |  | 63/70 |  |  |
| 3 |  |  | 17 |  |  | 85/85 |  |  |
| 4 |  |  | 17 |  |  | 83/95 |  |  |
| 5 |  |  | 18 |  |  | 72/75 |  |  |
| 1 |  | Δ*epsH* | 14 | 0.0018 | ** | 55/70 | 14.67  ± 0.67 | -20.5%  ± 0.9% |
| 2 |  |  | 16 | 0.0271 | * | 66/70 |  |  |
| 4 |  |  | 14 | 0.0261 | * | 86/95 |  |  |
| 2 |  | Δ*tasA* | 16 | 0.0161 | * | 78/78 | 15.33  ± 0.33 | -15.14%  ± 1.8% |
| 3 |  |  | 15 | 0.0026 | ** | 82/85 |  |  |
| 4 |  |  | 15 | 0.0307 | * | 91/95 |  |  |
| 2 |  | *tasA* (comp) | 18.5 | 0.8004 | ns | 80/80 | 17.75 ± 0.75 |  |
| 3 |  |  | 17 | 0.5781 | ns | 71/85 |  |  |
|  |  |  |  |  |  |  |  |  |
| 1 | N2 | wild-type**^t^** | 23 |  |  | 86/86 | 22.33  ± 0.67 |  |
| 2 |  |  | 21 |  |  | 79/80 |  |  |
| 3 |  |  | 23 |  |  | 86/86 |  |  |
| 1 |  | Δ*epsH***^t^** | 23 | 0.2299 | ns | 79/80 | 22  ± 1 |  |
| 2 |  |  | 21 | 0.303 | ns | 70/70 |  |  |
| 1 |  | Δ*tasA***^t^** | 23 | 0.433 | ns | 64/70 |  |  |
| 2 |  |  | 21 | 0.0646 | ns | 66/70 | 22.33  ± 0.67 |  |
| 3 |  |  | 23 | 0.433 | ns | 79/80 |  |  |
|  |  |  |  |  |  |  |  |  |
| 1 | *eat-2* | wild-type | 30 |  |  | 68/90 | 22.33  ± 0.67 |  |
| 2 |  |  | 34 |  |  | 41/70 |  |  |
| 3 |  |  | 35 |  |  | 70/100 |  |  |
| 1 |  | Δ*epsH* | 27 | 0.0434 | * | 51/80 | 22  ± 1 | -12.6%  ± 0.4% |
| 2 |  |  | 29 | 0.0341 | * | 42/70 |  |  |
| 3 |  |  | 32 | 0.0088 | ** | 65/89 |  |  |
| 1 |  | Δ*tasA* | 25.5 | 0.0002 | *** | 88/100 | 22.33  ± 0.67 | -28.6%  ± 9.1% |
| 2 |  |  | 28 | 0.0118 | * | 53/70 |  |  |
| 3 |  |  | 21 | < 0.0001 | **** | 68/90 |  |  |
|  |  |  |  |  |  |  |  |  |
| 1 | *mtl-1* | wild-type | 14 |  |  | 89/95 | 14.33  ± 0.33 |  |
| 2 |  |  | 15 |  |  | 65/75 |  |  |
| 3 |  |  | 14 |  |  | 91/91 |  |  |
| 1 |  | Δ*epsH* | 15 | 0.7362 | ns | 88/95 | 14.0  ± 0.5 |  |
| 2 |  |  | 15 | 0.1231 | ns | 67/75 |  |  |
| 3 |  |  | 13.5 | 0.0717 | ns | 90/90 |  |  |
| 1 |  | Δ*tasA* | 15 | 0.4988 | ns | 99/100 | 15  ± 0 |  |
| 2 |  |  | 15 | 0.2094 | ns | 75/75 |  |  |
|  |  |  |  |  |  |  |  |  |
| 1 | *daf-16* | wild-type | 12 |  |  | 84/85 | 12.5  ± 0.5 |  |
| 2 |  |  | 13 |  |  | 68/71 |  |  |
| 1 |  | Δ*epsH* | 12.5 | 0.6228 | ns | 76/77 | 12.25  ± 0.25 |  |
| 2 |  |  | 12 | 0.0614 | ns | 88/89 |  |  |
|  |  |  |  |  |  |  |  |  |
| 1 | *hsf-1* | wild-type | 16 |  |  | 97/97 | 15.5  ± 0.5 |  |
| 2 |  |  | 15 |  |  | 79/80 |  |  |
| 1 |  | Δ*epsH* | 15 | 0.0085 | ** | 67/67 | 14  ± 1 | -11%  ± 4.3% |
| 2 |  |  | 13 | 0.0132 | * | 67/68 |  |  |

Each data set (experiment #) was analyzed by building Kaplan-Meier survival curves and median survival time calculated. The percentage change in lifespan determined as an average of percentage change from each individual experiment with respect to biofilm-forming *B. subtilis* (wild-type) in the same experiment. Independent experimental and control analyses, which were performed side-by-side, are indicated by the same number (1, 2, or 3) in the first column. The log-rank test p-values (GraphPad Prism6) were calculated with respect to animals grown on wild-type *B. subtilis* in the same experiment.

^t^  - bacterial strains were treated with mixture of antibiotics (see Materials and Methods for details).

**Supplementary Table S4: Summary of worm size measurements.**

| Stage | *B. subtilis* wt | | | *B. subtilis* Δ*epsH* | | | *B. subtilis* Δ*tasA* | | | One-way ANOVA | |
| --- | --- | --- | --- | --- | --- | --- | --- | --- | --- | --- | --- |
|  | Mean, μm^2^ | SD, μm^2^ | N | Mean, μm^2^ | SD, μm^2^ | N | Mean, μm^2^ | SD, μm^2^ | N | *p*-value | Significance |
| L4 | 126003 | 31007 | 18 | 111234 | 26858 | 19 | 125828 | 10937 | 23 | 0.0917 | ns |
| A1 | 181583 | 47040 | 21 | 206697 | 48842 | 25 | 191710 | 47849 | 32 | 0.2056 | ns |
| A3 | 258199 | 45606 | 23 | 274093 | 63231 | 22 | 236468 | 53764 | 25 | 0.066 | ns |
| A4 | 345526 | 72598 | 28 | 359043 | 76265 | 27 | 351068 | 71660 | 29 | 0.7915 | ns |

**Supplementary Table S5: Differential expression of *C. elegans* genes in response to the *B. subtilis* biofilm.**

A list of *C. elegans* genes differentially regulated depending on the bacterial diet (q-value ≤0.05). Five-day old adult worms fed biofilm-forming *B. subtilis* NCBI3610 (wild-type) or Δ*epsH* strains were collected, total RNA was isolated and mRNA sequenced according to the Illumina True-Seq protocol. Differentially expressed genes were determined as described in Methods. Genes are sorted according to the statistical significance of their expression – the q-value, which is a p-value adjusted for multiple testing. Three independent biological replicates were sequenced.

**Supplementary Table S6: List of strains used in the study.**

| Bacterial strain | Genotype | Reference/Origin |
| --- | --- | --- |
| *B. subtilis* NCIB3610 | wild-type | 72 |
| DS76 | Δ*epsH*::tet | 72 |
| SSB505 | Δ*tasA*::spec | 73 |
| FC202 | Δ*tasA*::spec, amyE::PyqxM-yqxM-sipW-tasA | 73 |
| TMN503 | *sacA*::PtasA-mKate2 | 74 |
|  | PtasA-mKate2 ∆*sinI* | this study |
| *B. subtilis* CYBS-5 | wild-type | 17 |
|  | Δ*epsH*::tet |
| *P. fluorescens* Pf0-1 | wild-type | 68 |
|  | Δ*lapA* |
| *L. rhamnosus* GG (ATCC53103) | wild-type | 25 |
| CMPG5357 | Δ*spaCBA* |
| *E. coli* OP50 |  | Caenorhabditis Genetics Center, University of Minnesota |
| *C. elegans* strains |  |  |
| *C. elegans* N2 | wild-type | Caenorhabditis Genetics Center, University of Minnesota |
| PS3551 | *hsf-1(sy441)* I |
| CF1038 | *daf-16(mu86) I* |
| DA1116 | *eat-2* (ad1116) II |
| tm1770 | *mtl-1*(tm1770) | National Bioresource Project, Tokyo Women’s Medical University School of Medicine, Japan |
| WU1394 | pKD8 amEx183(Pmtl-1(WT)::MTL-1::GFP::mtl-1 3’UTR; myo-3::mCherry | 69 |
| *C. elegans* HT1593 | [*unc-119*](http://www.wormbase.org/species/c_elegans/gene/WBGene00006843)([*ed3*](http://www.wormbase.org/search/variation/ed3)) III | Caenorhabditis Genetics Center, University of Minnesota |
| *ilys-2* | ilys-2::unc-119 | this study |
| *hsp70* | PF44E5.4/5::unc-119 | this study |

**Supplementary Table S7: List of primers used in the study.**

**Supplementary Figures**

**Supplementary Figure S1:** **The beneficial effect of the *B. subtilis* biofilm is age-dependent.**

Each graph represents mean values ±SD from free independent biological replicates. Each biological replicate was performed with at least 60 worms per condition.

**a**, The biofilm does not increase thermotolerance in young adults. Day-one-adult worms grown on *B. subtilis* NCBI3610 (biofilm) or its biofilm-deficient mutants, Δ*epsH* and Δ*tasA,* were subjected to heat shock at 35°C for 4 hours. Surviving animals were scored after 20 hours of recovery at 20°C. One-way ANOVA p-value=0.9033. **b**, The biofilm does not promote heat resistance of *C. elegans* at day 3 of adulthood. Day-three-adult worms grown as described in (**a**) were subjected to heat shock at 33°C for 4 hours. Surviving animals were scored after 20 hours of recovery at 20°C. One-way ANOVA p-value=0.7163.

**Supplementary Figure S2:** **Biofilm formation by *B. subtilis* does not facilitate colonization of *C. elegans* intestine.**

Graphs show the scatter plots of colony forming units (CFU) per worm. Each data point in the column represents the result of independent biological replicate.

**a**, The amount of vegetative *B. subtilis* cells NCIB3610 (biofilm), biofilm-deficient mutants (Δ*epsH*, Δ*tasA*) and *E. coli* OP50 (OP50) in the *C. elegans* intestine at day 5 of adulthood determined by the colonization assay. For details see Material and Methods. Each data point in the column represents an average of the CFU per worm from 30 worms. One-way ANOVA p-value=0.5014 (for NCIB3610, Δ*epsH*, Δ*tasA*). **b,** The amount of vegetative *B. subtilis* cells (filled circles) and spores (clear circles) in the *C. elegans* intestine at day 5 of adulthood determined by guillotine method. For details see Material and Methods. Each data point in the column represents an average of the CFU per worm from 30 worms. Each data point in the column represents an average of the CFU per worm from 5 individual worms. T-test: p-value=0.7506 (vegetative cells count for NCIB3610 vs Δ*epsH*) and p-value=0.1554 (spores count for NCIB3610 vs Δ*epsH).*

**Supplementary Figure S3: *B. subtilis* biofilm extends *C. elegans* life span**.

**a**, Lifespan of *C.elegans* grown on wt *B. subtilis* NCBI3610 (biofilm)*,* biofilm-deficient mutant (Δ*epsH)* and *E.coli* OP50 (OP50). Mean lifespan, days: biofilm – 17; Δ*epsH* – 14; OP50 – 14. The graph is representative of two independent biological replicates. For the log-rank test p-values see Supplementary Table S3. **b**, Complementation of *B. subtilis* *tasA* deficiency restores biofilm-mediated enhancement of *C. elegans* lifespan. Worms were grown on either biofilm-forming *B. subtilis* NCIB3610 (biofilm), its biofilm-deficient derivative (Δ*tasA*), or ∆*tasA* complimented with a copy of *tasA* inserted at a distal locus (+tasA). Median lifespan, days: biofilm – 19; Δ*tasA* – 16; +*tasA* – 18.5. The graph is representative of two independent biological replicates. For the log-rank test p-values see Supplementary Table S3. **c**, The *B. subtilis* biofilm does not impair post-embryonic development timing and rate of egg production. **d**, The motility rate declines slower in biofilm-fed *C. elegans* between day 2 and days 8 of adulthood. Thrashing assay with *C. elegans* at day 2 or day 8 of adulthood grown on wild-type *B. subtilis* (biofilm) or biofilm deficient strains (Δ*epsH*, Δ*tasA*). The number of body bends per second per animal was measured as described in Materials and Methods. The graph represents values from 25 to 75 percentiles (Tukey whiskers), median and outliers.

**Supplementary Figure S4: The *B. subtilis* biofilm does not induce the dietary restriction response.**

**a**, Beneficial effects of biofilm are not induced by bacterial sporulation. *B. subtilis* NCIB3610 (biofilm) and its biofilm deficient mutant (Δ*epsH*) were grown on NGM essentially the same way as for *C. elegans* experiments. Sporulation rate was measured as described in Materials and Methods after 60 hours of growth, at which point worms would be normally transferred to the fresh plates. Mean values ±SD from three independent experiments are plotted, T-test *p*-value=0.36. **b**, Size of the worms at indicated developmental and adulthood stages grown on wt *B. subtilis* (biofilm) and biofilm mutants (Δ*epsH*, Δ*tasA*). The worm surface area was measured as described in Materials and Methods and mean values ±SD from three independent experiments are plotted. For statistical analysis see Supplementary Table S4. **c**, Rate of pharyngeal pumping of *C. elegans* grown on indicated bacterial strains. Pumping rate was measured as described in Materials and Methods. Table represents mean values from three independent experiments, where n=10 per experiment per condition. One-way ANOVA *p*-value= 0.5119.

**Supplementary Figure S5: Visualization of *B. subtilis* biofilm in *C. elegans* intestine.**

**
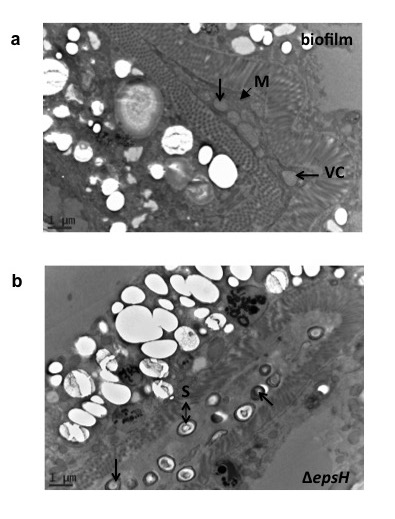
**

Transmission electron microscopy of the intestines of worms grown on the biofilm-forming *B. subtilis* NCIB3610 (**a)** or its biofilm-deficient derivative (**b)**. *B. subtilis* vegetative cells (VC) are marked by arrows, spores (S) by double arrows and dense matrix (M) by arrow heads.

**Supplementary Figure S6: Expression of target *C. elegans* genes in response to the *B. subtilis* biofilm.**

RT-PCR analysis of indicated genes in *C. elegans* grown on biofilm-deficient derivatives (Δ*epsH*, Δ*tasA*) as compared to biofilm-forming *B. subtilis* NCIB3610 at day 5 of adulthood. To calculate the fold change, the expression level of each gene in *C. elegans* fed wt *B. subtilis* was taken as 1. *C. elegans* genes with the statistically significant same change in expression under exposure to both biofilm mutants are marked with a star (p-value < 0.05). Expression analysis with RT-PCR was performed in three independent biological replicates and the mean value of fold change ±SE is plotted.

**Supplementary Figure S7: The role of DAF-16 and HSF-1 in biofilm-mediated effects**.

Lifespan of *C. elegans* CF1038 (daf-16(mu86)I) (**a**) and PS3551 (hsf-1(sy441)I) (**c**) fed NCBI3610 (biofilm) and its biofilm-deficient derivative (Δ*epsH*). Median lifespan of *daf-16* mutant, days: biofilm – 12; Δ*epsH* – 12.5. Median lifespan of *hsf-1* mutant, days: biofilm – 15; Δ*epsH* – 13. For each *C. elegans* mutant the graph represents two independent experiments. For the log-rank test p-values see Supplemental Table S3.

Thermotolerance of CF1038 (daf-16(mu86)I) (**b**) and PS3551 (hsf-1(sy441)I) (**d**) fed NCBI3610 (biofilm) and its biofilm-deficient derivative (Δ*epsH*). Five-day old worms were incubated at 33°C for the indicated time and survival was scored after 20 hours recovery at 20°C. Average values ±SD from three independent experiments are plotted. Each biological replicate was performed with at least 60 worms per condition. P-values were calculated for each time point using t-test.

**Supplementary Figure S8: Specificity of ILYS-2 in mediating biofilm-induced resistance to pathogenic infection.**

Representative Kaplan-Meier survival curves described in Fig. 1e, 6c and d.
